# Supplementary material for: Clinical perspectives on the identification of neurodevelopmental conditions in children and changes in referral pathways: qualitative interviews
Source: BMJ Open. 2022 Apr 13;12(4):e049821. doi: 10.1136/bmjopen-2021-049821 (PMC9014038; doi:10.1136/bmjopen-2021-049821)
Supplement: Supplementary data [file bmjopen-2021-049821supp001.pdf]

Case Study 1:
Reception received a phone call from patients Linda (33) and Tim (32) regarding an appointment for their son, Robert (6). The family are known to the practice and previously there have been safeguarding concerns and social services have been involved with the family.

In the initial phone call, Linda requested the next available appointment with the GP. They were subsequently booked in for an appointment in two weeks' time. The next day, Tim phoned reception to express his dissatisfaction with the waiting list and requested that they be given priority in the event of a cancellation.

Two weeks later Linda, Tim, and Robert arrived for the appointment. From the outset, Robert appeared distressed (i.e. crying). Linda made numerous attempts to comfort Robert, but he moved away in response to each of her approaches. At one-point Robert kicked out at Linda. There are what look like two distinctive episodes of hand-flapping.

As the consultation progressed, Robert gradually became more comfortable and was very active (e.g. jumping around the room). He moved from one activity to another in quick succession. Robert's eye contact was fleeting, and seemed to have a restricted range of facial expressions. In terms of conversation, Robert spoke in complex sentences, although the subject matter was a little repetitive and mainly around his favourite toy (Shopkins). Tim then took Robert to the waiting room, so Linda could discuss their concerns with the GP.

According to Linda, Robert has few friends in school and teachers are concerned about his academic progress. Additionally, Robert has become increasingly aggressive towards her and recently threw her laptop at a wall. In terms of history, she reports no significant issues with birth or pregnancy. Robert achieved his motor milestones; however, his language development was delayed. Previously, he received speech and language therapy in the community. Robert has an older half sibling, Chris (14) who has a diagnosis of ADHD. When asked about development prior to three years, Linda disclosed that Robert lived with his grandmother beginning when he was 18 months old to just after his third birthday, as Linda and Tim were separated during this period. During the separation, Linda was an inpatient at a local mental health facility.
