# Supplementary material for: Clinical perspectives on the identification of neurodevelopmental conditions in children and changes in referral pathways: qualitative interviews
Source: BMJ Open. 2022 Apr 13;12(4):e049821. doi: 10.1136/bmjopen-2021-049821 (PMC9014038; doi:10.1136/bmjopen-2021-049821)
Supplement: Supplementary data [file bmjopen-2021-049821supp002.pdf]

Supplement 2. Full study interview guide

Introduction:

I would like to ask you some questions about your training and background, your routine clinical activities, and your experiences and views on the diagnostic process for developmental conditions in children. I would like to remind you not to disclose any personally identifiable information about any individuals, child or families you work with during the interview.

Background
Can you give me a brief overview of your current position?
Probe for experience related to child development

How long have you been working as a (GP, psychologist, psychiatrist etc)?
Probe for length of time working with children and families

Where and when did you do your clinical training?
Probe for General Practitioner (GP) training vs medical training

Clinical work

Without revealing any personally identifiable information, can you tell me about a case where you conceptualised the child's features as relating to autism?
Probe action steps
Probe specific difficulties with working with this child
Probe for informal assessment (e.g. familiarity with things in the past)
Probe assessments (I know X is saturated with screening tools/assessments; observations)
Probe for level of clarity (how certain or uncertain were you about X)
Repeat for ADHD and attachment related

If not clear, can you give me an overview of your involvement in relation to identification and assessment of developmental (or behavioural) difficulties?
Probe for assessment tools, observations and focus on developmental histories, team-based approach etc.
If GP, probe for screening tools.

Can you think of a specific instance where there was uncertainty regarding the nature of a child's difficulties/diagnosis?
Probe for description
Probe for action steps

Are there other factors that contributed/routinely contribute to the decision you made (e.g. system factors, availability of supports and services)?
Have you ever been involved with overturning or changing a child's diagnosis?

Case Study Questions

Questions for participants with Case Study 1,

What are your initial thoughts on the case?
What questions would you ask A) Robert B) Parents (Tim & Linda)
What would you advise as the next steps?
If the participant deems onward referral is necessary
what information would you put in the referral letter?
Why did X resonate with you?
How easy have you found it to get external help

What did you find yourself drawing on to make sense of Roberts presenting difficulties? (i.e. other similar cases, diagnostic criteria, theory etc)

If the child had a private diagnosis of autism, would that influence how you think about the case?

If you were unsure about the nature of the child's difficulties, are there other professionals you would consult with?

Questions for participants with Case study 2.

What are your initial thoughts on the case?
Probe for possible diagnosis, working clinical hypotheses, formulation

If the participant states a specific diagnosis/conceptualisation
What features of the case helped you to arrive at that conclusion?
Are there other conditions you considered?
Probe, if so what?
Probe how the participant differentiated

If the participant indicates that it could be multiple conditions
You mentioned that it could be X or Y, how would you differentiate?
What assessments/sources of information would be useful in helping you to reach your decision?

If the participant indicates there isn't enough information to make the decision
What information would help you make a decision regarding the nature of the child's difficulties?
Are there specific assessments/ theories you would use?

In your view, is there need for further assessment?
If Yes, what are the next steps in terms of assessment?

Probe for risk assessment
Probe cognitive assessment
Probe sources of information

Are there any frameworks/ theories you used to understand the nature of the child's presenting difficulties?

Probe for specific models/ theories
Probe for influential book/text/ or talks

If the child had a private diagnosis of autism, would that influence how you think about the case?

If you were unsure about the nature of the child's difficulties, are there other professionals you would consult with?
Referral Pathways Information

In your experience, what do you consider the early markers of autism?
Probe for symptoms, features, predisposing events, comorbid conditions, family history
**repeat for ADHD, Attachment related difficulties.

Rotate order each interview

Questions for Primary Care Clinicians
Have you ever referred a child to a CAMHS or relevant assessment service as you suspected the child may a developmental condition or behavioural difficulty?
If yes, what information did you include in your referral letter?
Have you ever had a referral of this nature not accepted in the first instance?
If yes, why was the referral not accepted?
On a scale of 1-10, how easy have you found it to get formal assessment for a child you think refer a child who you think may have autism?
Repeat for attachment problems
Repeat for ADHD
Have you experienced any challenges to referring a child who you think may have autism?
Repeat for attachment problems
Repeat for ADHD
What, if anything could be done to make the referral pathway, clearer?
On a scale of 1-10, how confident do you feel that you will be able to get the appropriate a) assessment b) support for a child with autism, adhd, attachment problems.

Questions for non-primary care clinicians
Do you receive many referrals to your service from GPs?
If yes, how would you describe the quality of these referrals?
Probe what's in a good/less good referral
If no, where do you get the majority of referrals from
How would you describe the quality of these referrals?
What information would constitute a sufficient referral (i.e. allows you to make your decision regarding acceptance for subsequent assessment/support)
What information could be collected by a GP to assist with the decision to accept or reject a referral to your service?
How do you think children and families experience the journey from primary care to your service?
Thinking about conditions with overlapping features, do you see value in differentiating autism from attachment-related conditions?
Do you see a meaningful distinction between the attachment disorders (RAD and Disinhibited) and attachment-related difficulties?

Similarly, when considering a diagnosis of autism and adhd, what value do you see in diagnosing both or trying to differentiate?
Finally, what in you view is the value in differentiating ADHD from attachment-related conditions?
