# Supplementary material for: Clinical perspectives on the identification of neurodevelopmental conditions in children and changes in referral pathways: qualitative interviews
Source: BMJ Open. 2022 Apr 13;12(4):e049821. doi: 10.1136/bmjopen-2021-049821 (PMC9014038; doi:10.1136/bmjopen-2021-049821)
Supplement: Author's manuscript [file bmjopen-2021-049821.draft_revisions.pdf]

BMJ Open

BMJ Open is committed to open peer review. As part of this commitment we make the peer review history of every article we publish publicly available.

When an article is published we post the peer reviewers' comments and the authors' responses online. We also post the versions of the paper that were used during peer review. These are the versions that the peer review comments apply to.

The versions of the paper that follow are the versions that were submitted during the peer review process. They are not the versions of record or the final published versions. They should not be cited or distributed as the published version of this manuscript.

BMJ Open is an open access journal and the full, final, typeset and author-corrected version of record of the manuscript is available on our site with no access controls, subscription charges or pay-per-view fees (http://bmjopen.bmj.com).

If you have any questions on BMJ Open's open peer review process please email info.bmjopen@bmj.com

BMJ Open

Clinical perspectives on the identification of neurodevelopmental conditions in children and changes in referral pathways: Qualitative interviews.

Journal:	*BMJ Open*
Manuscript ID	bmjopen-2021-049821
Article Type:	Original research
Date Submitted by the Author:	03-Feb-2021
Complete List of Authors:	Coughlan, Barry; University of Cambridge, Department of Public Health and Primary Care<br>Woolgar, Matt ; King's College London Institute of Psychiatry Psychology and Neuroscience<br>Mann, Alissa; University of Bath, Department of Psychology<br>Duschinsky, Robbie; University of Cambridge Primary Care Unit
Keywords:	Community child health < PAEDIATRICS, Developmental neurology & neurodisability < PAEDIATRICS, PRIMARY CARE, Child & adolescent psychiatry < PSYCHIATRY, QUALITATIVE RESEARCH

SCHOLARONE™
Manuscripts

I, the Submitting Author has the right to grant and does grant on behalf of all authors of the Work (as defined in the below author licence), an exclusive licence and/or a non-exclusive licence for contributions from authors who are: i) UK Crown employees; ii) where BMJ has agreed a CC-BY licence shall apply, and/or iii) in accordance with the terms applicable for US Federal Government officers or employees acting as part of their official duties; on a worldwide, perpetual, irrevocable, royalty-free basis to BMJ Publishing Group Ltd ("BMJ") its licensees and where the relevant Journal is co-owned by BMJ to the co-owners of the Journal, to publish the Work in this journal and any other BMJ products and to exploit all rights, as set out in our licence.

The Submitting Author accepts and understands that any supply made under these terms is made by BMJ to the Submitting Author unless you are acting as an employee on behalf of your employer or a postgraduate student of an affiliated institution which is paying any applicable article publishing charge ("APC") for Open Access articles. Where the Submitting Author wishes to make the Work available on an Open Access basis (and intends to pay the relevant APC), the terms of reuse of such Open Access shall be governed by a Creative Commons licence – details of these licences and which Creative Commons licence will apply to this Work are set out in our licence referred to above.

Other than as permitted in any relevant BMJ Author's Self Archiving Policies, I confirm this Work has not been accepted for publication elsewhere, is not being considered for publication elsewhere and does not duplicate material already published. I confirm all authors consent to publication of this Work and authorise the granting of this licence.

For peer review only

Clinical perspectives on the identification of neurodevelopmental conditions in children and changes in referral pathways: Qualitative interviews.

Barry Coughlan[1], Matt Woolgar[2], Alissa Mann,[3,] Robbie Duschinsky[1]

[1]Department of Public Health and Primary Care, University of Cambridge, Cambridge, UK

[2] Institute of Psychiatry, Psychology and Neuroscience, King's College London, London, UK

[3]Department of Psychology, University of Bath

Corresponding author: Mr Barry Coughlan (bc471@medschl.cam.ac.uk); Telephone: 00353830797072

B Coughlan is a PhD candidate and Research Associate. ORCID: 0000-0002-1484-6491

M Woolgar DClinPsy and PhD Consultant Clinical Psychologist; matt.woolgar@kcl.ac.uk; ORCID: 0000-0002-3618-0395

A Mann is a BSc Psychology student at the University of Bath; afm46@bath.ac.uk

R Duschinsky Head of the Applied Social Sciences Group, Department of Public Health and Primary Care at the University of Cambridge; rd522@medschl.cam.ac.uk; ORCID: 0000-0003-2023-5328

Conflicts: No conflicts to declare

For peer review only - http://bmjopen.bmj.com/site/about/guidelines.xhtml

For peer review only

Clinical perspectives on the identification of neurodevelopmental conditions in children and changes in referral pathways: Qualitative interviews.

Word Count = 4552

Abstract

Background: Previous work has raised questions about the role of General Practitioner's (GPs) in the identification of neurodevelopmental conditions such as autism spectrum disorders (autism) and attention deficit hyperactivity disorders (ADHD). This work has tended to foreground certain forms of knowledge (i.e. knowledge of clinical markers) whilst backgrounding other forms of information.

Objective: This study aimed to explore how GPs identify these conditions in practice and their perspectives on recent changes to local referral pathways that mean that GP referrals are rarely accepted. We also aimed to explore specialists views on the role of GPs.

Method: Semi-structured interviews were conducted with GPs (n=8), specialists in local services (n=7), and professionals at various specialist services around the country (n=10). Interviews were conducted between January and May 2019. A framework approach informed by thematic analysis was used to analyse the data.

Results: GPs drew on various forms of tacit and explicit information including behavioural markers, parental report, prior knowledge of the family, expert and lay resources. Opinions varied between GPs regarding changes to the referral pathway, with some accepting the changes and others describing it as a "disaster". Specialists tended to feel that GPs required more neurodevelopmental training and time to conduct consultations.

Conclusion: This study adds to the literature showing that GPs use an array of sources of information when making referral decisions for autism and ADHD. Further work is urgently required to evaluate the impact of reconfiguring neurodevelopmental referral pathways such that GPs have a diminished role in identification.

Keywords: Autism, ADHD, General Practice, Referral pathways

For peer review only - http://bmjopen.bmj.com/site/about/guidelines.xhtml

For peer review only

Strengths:

- This study uses qualitative interviews and case study approach by doing some shines important light on GP decision-making processes and perspectives on changes referral pathways
- The interview schedule was extensively piloted with various professionals prior to data collected and generated rich data
- Data analysis had inductive and deductive elements building from previous review work

Limitations

- GP were recruited through the local CRN and thus we are not able to establish whether non-CRN GPs who have differing perspectives
- This work is not epistemologically or methodological positioned to comment on the effectiveness of the referral pathways.

Introduction

Gatekeeping - the act of determining access to specialist care and diagnostic services - is a routine task for GPs. A core goal of the gatekeeping model is to make healthcare accessible while ensuring that service delivery is feasible. Concerns about the effectiveness of GP gatekeeping are longstanding in the primary care literature (e.g. 1, 2, 3). Recent reviews have suggested that, in general, GP gatekeeping is linked with a better quality of care and lower service utilisation (4). Yet questions persist about patient satisfaction with the model and the accuracy of gatekeepers in identifying certain conditions (e.g. 4). In the UK, some clinical commission groups have alleviated GPs of their gatekeeping responsibilities for specific clinical populations, including paediatrics and some mental health services (5). This has been done by shifting gatekeeping duties to professionals in adjacent fields (e.g. health visitors, social care, and education) or introducing direct referral or self-referral models.

The assessment of developmental conditions such as autism spectrum disorders (autism) and attention deficit hyperactivity disorders (ADHD) reflect these broader tensions around the gatekeeping role in primary care. Referral pathways in the UK often require that GPs initiate referrals for children where there is a query of autism or ADHD. Much of the research on autism and ADHD in general practice focuses on GP knowledge and attitudes towards the respective conditions (6-9). Survey work indicates that GPs have a sound understanding of

For peer review only - http://bmjopen.bmj.com/site/about/guidelines.xhtml

autism, but little confidence responding to the condition (7). Still, review work on GPs knowledge of autism and ADHD have identified some outmoded aetiological theories still receiving endorsement (6, 10). Consequently, calls for training, from GPs and researchers alike, are recurrent in much of this work.

Remarkably few studies, however, have explored how GPs make these decisions in practice. This is within a context where parents often describe the pathway to diagnosis as challenging (11-13), and reasons for delays in referral are often felt by parents to be unclear. Some insight, however, can be gleaned from a Canadian study by Kennedy, Regehr (14) on medical students at the University of Toronto on knowledge-practice discrepancies following educational programmes. In this qualitative study, the authors identified an array of factors including patient motivations, systemic issues, social and clinician factors as explanations for referral decisions. Increased uncertainty and urgency, somewhat predictably, prompted referrals (14). Indeed, clinical judgment appears to be an essential factor even within contexts where best practice guidance recommends standardised screening for developmental conditions (15). For instance, a randomised controlled trial of physicians comparing standard assessments versus traditional methods (i.e. clinical judgment and developmental milestones) found higher detection rates in the group using standardised assessments, and yet referral rates did not significantly differ (16). This finding prompted the authors to conclude that clinical judgment still overrides standardised assessments.

Our study sought to broaden the understanding of how GPs identify developmental conditions such as autism and ADHD in practice. Moreover, our research takes place in an English city where changes to the configuration of local pathways mean that referrals from GPs are rarely accepted. Therefore, a subsidiary aim was to explore how GPs experience these changes and also how clinicians in specialist services think about the role of GPs. Although this study takes place in a particular setting, the themes identified here will have relevance to broader national conversations about the organisation of referral pathways and the gatekeeping role of GPs.

Method

The data presented in this study were collected as part of a project exploring assessment practices in health care professionals (n=25). Specifically, we conducted semi-structured interviews with eight GPs and seven healthcare professionals working across a

For peer review only - http://bmjopen.bmj.com/site/about/guidelines.xhtml

neurodevelopmental team, and child and adolescent mental health services (CAMHS) in an English city. Additionally, we interviewed a further ten professionals who were working at various social and neurodevelopmental services, including tier 4 national services, across the UK. Here we report on the part of the study concerned with GPs experiences of identifying autism and ADHD, changes to local referral pathways, and the views of specialists regarding the role of GPs in the neurodevelopmental assessment. This project was approved by the University of Cambridge Psychology Ethics Committee [PRE.2018.019], The Health Research Authority and local NHS research and development teams. All participants provided written informed consent before to data collection. Consent was also provided verbally at the end of each interview.

Referral Pathway

The study was conducted in a socioeconomically, diverse English city. Here community and paediatric teams often work together to provide services for children under five years with a suspected developmental condition including autism and ADHD. This referral pathway is configured such that referrals mostly come from preschools and or health visitors. For school-aged children, referrals tend to go through schools unless the child has an established neurodevelopmental condition. In the first instance, most parents are offered support. Should questions remain about the child's development, then an assessment is conducted by the neurodevelopmental team. Community CAMHS, on the other hand, work with children with mental health problems and accept a referral from an array of sources including GPs, allied healthcare professionals, social workers, and education professionals. There are also teams specialising in child safeguarding.

Data collection

The local clinical research network invited GPs to take part. Professionals from CAMHS, social and neurodevelopmental services were recruited using a combination of purposive, convenience, and snowball sampling techniques. BC conducted all interviews either in person or remotely (e.g. via telephone). Face-to-face interviews were conducted in GP practices or clinic rooms. Data were collected between January and May 2019. For further information, see Table 1. Before data collection, we developed a topic guide based on existing literature and experience of the authors. The guide was piloted with three healthcare professionals working in mental health or developmental services. Questions were also discussed with two academic GPs. The final version of the guide was divided into the following sections: professional

For peer review only - http://bmjopen.bmj.com/site/about/guidelines.xhtml

background, routine clinical work, a hypothetical case study, and referral pathways. See supplement (S1) for the hypothetical case study. At the beginning of each interview, participants were asked not to disclose any personally identifiable information about any patients. Questions in the section on routine clinical work were also prefaced with this reminder.

BC has experience working in a neurodevelopmental service as an assistant psychologist. Here he became interested in the interaction between cognate health services. MW is a consultant clinic psychologist and RD is a social scientist. Both RD and MW are interested in assessment practices for social and neurodevelopmental conditions. AM is a placement student with an interest in child development.

We adopted an 'information power' approach to guide recruitment and sample size (17). This approach spotlights the following considerations for establishing a sample size in qualitative research: study aim; sample specificity; established theory; quality of dialogue; and analysis strategy (17).

Patient and Public Involvement

A general patient and public review panel at a local hospital provided feedback and suggestions on the research materials, including the topic guide. To flag, this panel did not necessarily have experience or personal contact with ASD or ADHD.

Data analysis

Data were analysed and interpreted using the framework method outlined by Gale, Heath (18). This method has the advantage of inductive and deductive elements. This, therefore, allows for ideas from the existing literature to be brought together with data derived from the interviews to develop an analytical framework. This included a recent systematic review on autism in general practice (10) and a review by Tatlow-Golden and colleagues on GPs and ADHD. All interviews were transcribed by BC or professional transcription service. Transcripts were read three times, and all audio recordings were listened to at least once before the first round of coding. In the initial stages, transcripts were coded using line-by-line coding. All transcripts were coded by BC, and several of the transcripts were also read in full by AM and RD. Regular meetings were held between the authors to discuss the data. All authors approved the final series of themes. Transcripts were coded by hand, and data were organised and grouped using flashcards. Here results pertaining to identification and referral pathways are discussed.

For peer review only - http://bmjopen.bmj.com/site/about/guidelines.xhtml

Specialist neurodevelopmental assessment practices and differential conceptualisation are explored elsewhere. Prior to submission, participants were each sent the results and offered the opportunity to comment on the findings.

Table 1: Participant and interview characteristics

Participant ID	Gender	Experience (years)	Setting	Interview Length
**PTGP01**	Female	>20 years	Local GP practice	43 mins
**PTGP02**	Male	4 years	Local GP practice	41 mins
**PTGP03**	Female	>20 years	Local GP practice	44 mins
**PTGP04**	Male	>20 years	Local GP practice	64 mins
**PTGP05**	Male	19 years	Local GP practice	29 mins
**PTGP06**	Male	>20 years	Local GP practice	37 mins
**PTGP07**	Male	>20 years	Local GP practice	71 mins
**PTGP08**	Male	14 years	Local GP practice	61 mins
**PTND01**	Male	17 years	Local ND service	66 mins
**PTND02**	Female	>20 years	Local ND service	64 mins
**PTND03**	Female	13 years	Local ND service	58 mins
**PTND04**	Female	> 20 years	Local ND service	64 mins
**PTND05**	Female	14 years	Local CAMHS	69 mins
**PTND06**	Female	13 years	Lifespan Autism Service	65 mins
**PTND07**	Male	3 years	Child autism service	55 mins
**PTND08**	Female	10 years	Tier 4 CAMHS	62 mins
**PTND09**	Female	19 years	Tier 4 CAMHS	53 mins
**PTND10**	Female	10 years	Tier 4 CAMHS	58 mins
**PTND11**	Female	16 years	Tier 4 CAMHS	48 mins
**PTND12**	Male	6 years	Tier 4 CAMHS	54 mins
**PTND13**	Female	>20 years	Tier 4 CAMHS	43 mins
**PTND14**	Male	>20 years	Tier 4 CAMHS	55 mins
**PTND15**	Female	4 years	Tier 4 CAMHS	61 mins
**PTND16**	Male	4 years	Local CAMHS	63 mins
**PTND17**	Female	>20 years	Local ND	65 mins

ND = Neurodevelopmental, CAMHS = Child and Adolescent Mental Health Services

Results

We will present our findings in two sections. The first focuses on the methods, and sources of information the GPs (n=8) used when identifying autism and ADHD in children. The second section discusses material from the entire set of transcripts (n=25) to explore a range of perspectives on changes to the pathway and the role of the GP.

Identification

For peer review only - http://bmjopen.bmj.com/site/about/guidelines.xhtml

There was some variation regarding the methods and techniques used by GPs to identify autism and ADHD in children. References to a diverse array of forms of information could be seen across the transcripts, including both tacit to explicit sources. These include various clinical or behavioural markers, unstructured behavioural tasks, prior knowledge of the family, and professional networks and personal experience. Nevertheless, the extent to which GPs considered, used, and triangulated this information varied considerably.

Explicit information: An assortment of diagnostic or clinical markers for each condition were described by participants. That is, practitioners often made reference to specific traits they considered features of certain conditions. Yet some GPs expressed uncertainty and hesitancy when asked about particular indicators:

"Early markers? I'd probably have to look it all up, actually…And often I do. When I've got a patient coming in, I just have a sort of screen what the most common symptoms" PTGP02

"There's gonna[sic] be diagnostic criteria for that but don't ask me what they are. There's a big long list of diagnostic criteria, but I kind of think that's more a specialist job to apply the diagnostic criteria in detail before making the diagnosis, but I'd probably spot the warning signs as it were and refer on as appropriate." PTGP06

And indeed, several practitioners described looking up markers using professional sources such as GP Notebook, Clinical Knowledge Summaries or Patient.co.uk as well as some lay sources including Google or Wikipedia to find specific behavioural markers.

In general, however, GPs appeared to agree on the importance of parental report. This is, of course, understandable as parental concerns are an essential component of the formal assessment for many behaviourally diagnosed developmental conditions. While describing past cases, one GP commented:

"Nine-tenths is the story you're given by the parents. Because they are the… as I say to parents, you know your son or daughter better than anybody in the world. So, we have to listen to what they have to say, [and their] ideas, concerns, and expectations" PTGP07

For peer review only

And indeed, the majority of participants expressed similar sentiments. Importantly, however, most GPs indicated that parental report alone was not sufficient grounds for a referral. Instead, it was suggested that such reports should be corroborated with observations of the child. Yet when facing uncertainty, approaches varied. For instance, after reflecting on complex or uncertain cases, one GP remarked:

"Just got to go with what the parents are thinking" PTGP06

However, another GP was especially concerned with the threat of overdiagnosis. For this GP, it was particularly important to triangulate parental concerns, observations of the child, and reports from the child's school. This GP reflected on a case where parents queried a diagnosis of ADHD following conversations with a family friend:

"Speaking to the friend caused them to say maybe he [the child] has got ADHD. But in actual fact, I really don't think he has, and the last thing you'd want is for this kid to go on unnecessary medication" PTGP04

He went on to explain that after receiving consent from the child's parents to contact the child's school:

"[I] spoke to his teacher and actually this was an example of where the school actually had a really good handle on him. The teacher said he's a lovely kid, but he's essentially feral. He just isn't set up for rules so there was nothing he's doing at school that would make me worried. He's a lovely lad, and you can engage him, and he can concentrate and focus when he wants to" PTGP04

In contrast, however, there was a least one instance where a GP's decision to refer seemed to be based predominantly on parental insistence, rather than clinical observations or judgment:

"[Refers to another family member] seemed to know it all. [Parent] was saying that they thought the child had autism on the basis that [the child is] behind with learning, not reading and writing yet, didn't like social situations…[…]…. And they said that the school didn't think the child had autism. So, I have referred... I mean [the child] seemed normal, sat doing not a lot, but seemed normal." PTGP05

For peer review only - http://bmjopen.bmj.com/site/about/guidelines.xhtml

For peer review only

Subsequently, this participant indicated that the chances of the referral being rejected were '100%' due to the configuration of local referral pathways. When this happens, he explained he would urge the parents to go back to the school.

Tacit information: GPs also often drew implicitly from the language of folk psychology regarding typical and atypical child development. Phrases such as 'a little odd', 'just isn't what most children do" or 'clashes with normal expectations' can be found throughout the data. These were often used in reference to a specific marker or behaviour, such as "rituals and behaviours that weren't quite in keeping with a normal child of her age". Here the term tacit knowledge is used broadly to refer to practical or soft knowledge that is not easily quantifiable.

Clinical intuition was important for deciding between typical and atypical development, but at times, challenging to articulate:

"As a GP you get a subconscious idea of the spectrum of the range with children - from the kid who'll sit there like butter wouldn't melt in their mouth, like a bit oddly so, to the kid who's climbing up your curtains. [And] You get a feel of parental interaction, with 'you stop doing that now I've told you before' to the parent who just watches the child smash your ophthalmoscope'" PTGP04

"I think it's difficult, sometimes, to describe what turns into a kind of sixth sense. Really you get a clue, don't you? And sort of that kind of gut feeling, but it is about the behaviour." PTGP07

Prior knowledge or experience with specific children and families was also crucial for several GPs. When reflecting on cases, it was not uncommon for practitioners to preface conceptualisations with remarks such as 'I've known him since... well antenatally', 'I know the family' or '[Mum/Dad] is also my patient'. This seemed to offer a degree of context and explanation for the child's presentation. For instance, when describing children with a query of a neurodevelopmental condition, some GPs remarked on traits they had seen in other family members or diagnoses of other family members there were aware of.

For peer review only - http://bmjopen.bmj.com/site/about/guidelines.xhtml

GPs were also attuned to socio-environmental or parenting factors that might be contributing to the child's presenting symptoms such as discrete participating events, parental separation or conflict. Having this overview of the patient was, for many, one of the core strengths of general practice:

"I suppose this is where Family Medicine really comes into its fore, isn't it? Because they're [both child and parents] usually, not always, but usually all our patients. So, sometimes we have this interesting dilemma about whom is the patient." PTGP07

Yet this expertise, some felt, was not always appreciated by colleagues in specialist services. When reflecting on the experience of having referrals rejected, one GP remarked:

"I sometimes wonder whether they [specialist assessment service] actually consider the family factors that we know of that we write in out letters" PTGP03

Perceptions of the new referral pathway

Most of the GPs were aware of the changes to the assessment pathway that meant referrals for neurodevelopmental assessment typically come through schools or health visitors. However, it was unclear whether two GPs were aware of these changes. Three stances to these changes were identified in the data: accepting, ambivalent, and critical. Practitioners who were more accepting of the changes trended to reason that schools are better positioned to identify such developmental conditions:

"Well, you see I think community paediatrics probably has a point. Because small child gets brought in to see the doctor and they're looking around looking reasonably normal but what do I know. Whereas the school and other people that interact with the child over a long period of time are in a better position to make an assessment than me." PTGP05

"They'll [schools] be better at recognising it than me, so I'm happy, doesn't matter where the referral comes from, as long as it happens in a timely way it doesn't have to come from a GP." PTGP06

For peer review only - http://bmjopen.bmj.com/site/about/guidelines.xhtml

One participant had a more ambivalent attitude. For this participant, there was an acknowledgement that schools are often well-placed to identify atypical development. Still, they maintained that limiting the ability of GPs to refer put them in a challenging position:

“Difficult. Because you can see the logic in that, actually there’s so much more to this than having a name put to your child’s odd behaviour very few of these children will benefit from something medical…[…]… the problem comes really when a parent comes in and says I’ve been to the teacher three times, and the teacher says they think he’s fine and if you’re really worried you can go and see your GP. Because you’ve no idea did the teacher really say that.” PTGP04

While one GP was critical of the pathway:

“Just a disaster, just a road crash really - trying to get children seen with developmental or behavioural problems is increasingly difficult, and in fact, for many patients, we end up having to go if they’re school age we end up having to go through school…[…]... And that’s a real nightmare for me because it means I’m having to delegate that to a third party who is not actually a health service” PTGP07

This GP felt that the pathway was also a threat to professional status and identity, reflecting a devaluation of primary care.

Professionals in the neurodevelopmental services tended to view the changes as positive. Professionals in the neurodevelopmental team reported the impact these changes have had on service-level pressures, including waiting times for assessment:

“We’ve got the shortest waiting times for assessment for autism and ADHD. Less than eighteen weeks, whereas they were eighteen months to two to three years [before]” PTND01

Specialist views on the role of GPs

When asked directly whether GPs had a role in identifying developmental conditions, most specialists indicated that there was indeed a role for GPs. Yet this was often couched with an array of caveats about professional and organisational barriers to identification. The most

For peer review only - http://bmjopen.bmj.com/site/about/guidelines.xhtml

common barrier, according to the specialists, was the duration of primary care consultations and a lack of training or knowledge about neurodevelopmental conditions:

“They need to be given more time to do it properly and more training. They get very little training at all really but if they got proper training and given a bit more time. Even fifteen-twenty minutes, but at the moment all they could do is to at least know the NICE guidance and know what are the signs and symptoms and take a detailed history and follow the local pathway really. Clearly, if we have GPs with a special interest in children, they got better training, and clearly, they have a lot of role to play with the ADHD medication shared care and those kinds of things.” PTND01

“At best what they should do is make good referrals to specialist teams. But beyond that, I don’t know if it would be useful for people who are under massive strain and pressure and who have like whatever is it eight to ten-minute appointments, I hear that’s the average, but I’ve never had any more than six minutes really, so I mean I don’t know how you could do anything bar account for the family’s request and signpost them to the appropriate teams.” PTND07

A lack of training was also framed as problematic by one GP:

“I think also in terms of what we get taught it may change now obviously I trained thirty years ago literally we had no training at all...[…]… We’d all heard of autism but everything I know about neurodevelopmental disorders, not that there’s much of it, has been acquired post-grad.” PTGP04

References to the time afforded for consultations can also be found throughout the GP interviews. For some, this was felt to be a significant barrier to identification. To circumnavigate some of these challenges, one GP described bringing families back for multiple consultations.

Information sharing as a barrier and opportunity:
Another topic that runs through the data is the importance of informal networks and the issues with sharing information between services. In general, informal networks could be described as internal and external. Internal networks mostly consisted of practice staff, including administrative staff, GP colleagues and nurses. By contrast, external networks consisted of

For peer review only - http://bmjopen.bmj.com/site/about/guidelines.xhtml

educational professionals and colleagues in secondary care. Due to the reconfiguration of primary care services, health visitors seemed to occupy a position between these two networks:

"We used to have Health Visitors attached to the practice, but they don't exist anymore. I don't know who our Health Visitor is I've never met them." PTGP05

Meanwhile, most GPs acknowledged that nursery staff, primary school teachers and other educational professionals were essential sources of information when thinking about child development. Yet the lack of a linked system for educational and primary care records presented challenges in terms of sharing this information:

"We have occasional contact with schools but not very much. Not often. I'd be unsure about the boundaries and confidentiality and things like that, to be honest." PTGP05

There also seemed to be a lack of communication between GPs and specialist services:

"It's so difficult because you know you'll write the letter, but you don't know if they'll actually get any help or whether they'll get put on the waiting list or whether someone else will monitor the child. So that's the tricky bit really." PTGP03

"let's say we're querying autism they [neurodevelopmental team] would send the referral back. And say it needs to be referred through the school which is quite doable because quite often they have started with the school. And the school have said have you seen your GP and of course then it looks like passing the parcel." PTGP04

For peer review only

Discussion

Summary

GPs used tacit and explicit forms of information when identifying autism and ADHD in children. These included clinical or behavioural markers, parental report, prior knowledge of the child and family, and professional networks. For most, parental concerns were the chief factor driving referral decisions. A few participants, however, described instances where they had sought information from other sources (e.g. schools). Nevertheless, changes to the configuration of local pathways have meant that referrals from GPs for neurodevelopmental

For peer review only - http://bmjopen.bmj.com/site/about/guidelines.xhtml

assessment are now rarely accepted. GPs had mixed views on these changes. Most specialists agreed that GPs did have a role in identifying neurodevelopmental conditions yet expressed concerns about a perceived lack of training or knowledge and framed the length of time as problematic.

Strengths and Limitations

The current study adds to our understanding of early identification by gleaning the perspectives of GPs and those in specialists assessment services. From a methodological perspective, the flexible interview guide and the combination of case-based discussions and clinical vignette allowed us to elicit rich narratives about these topics. Further, by analysing discourses of past and hypothetical cases, we were able to explore some of the other forms of knowledge that come into play. Additionally, our study was conducted in a setting where GPs have been, to a large extent, absolved of their gatekeeping responsibilities for identifying autism and ADHD in children. Therefore, the current study presents a unique opportunity to explore how GPs experience having a reduced role for a specific patient group and thus adds to national conversations about the nature and future of general practice. That said, it is essential to consider whether the findings about identification are transferable to other contexts. Regarding identification, given that specialists espoused similar issues with referrals in different settings, it seems unlikely that the methods and techniques used by GPs in this area were atypical. As recruitment of GPs was completed through the local CRN, it is not possible to determine how many GPs decided not to take part in the study. This might raise other concerns about the representativeness of the GP sample. However, as the analysis illustrates, there was considerable diversity in the views and opinions expressed by the GPs.

Comparison with the literature

Most studies about GP knowledge of autism and ADHD have focused on explicit knowledge of clinical markers (6, 7, 9, 19). Yet as others have shown clinical judgment is core to referral decisions (14, 16). Naturally, knowledge of clinical markers is important for identifying these conditions. Yet, an overemphasis on this form of knowledge risks driving attention away from the other sources GPs draw on including prior experience with the child or family. Our study, therefore, adds to the understanding of identification by tracing out the various forms of explicit and tacit material, which GPs draw upon when determining whether a child requires formal assessment.

For peer review only - http://bmjopen.bmj.com/site/about/guidelines.xhtml

Several studies have identified that GPs frequently report having little training in autism (e.g. 7) and ADHD (6). It follows that more training is needed. Our data lend some support to these findings and broadly speaking, we agree with these calls for more training. The 'lack of training' thread runs through the primary care literature. Yet a degree of caution is warranted as framing the problem as one of 'a lack of training' risks a) flattening the conceptual complexity associated with identifying these conditions b) silencing the host of organisational shortcomings that make referral decisions challenging, and c) camouflaging alternate solutions such as the integration of health, educational, justice or social care records or changing pathways.

Implications for Research and Practice

Elsewhere questions have been raised about GP gatekeeping (1-3). As such, zooming in on a particular pathway means that we were able to explore in detail how those on the ground experienced changes to GP gatekeeping. It might be envisaged that GPs would welcome changes that reduce some of the pressure on them. Yet GPs in this study expressed mixed views. In contrast, specialists tended to view the changes positively and credit these changes with preventing saturation of the service. Our research is not positioned to explore the impact that these changes have on service delivery. We recommend that future work explores how such changes impact patient satisfaction, waiting lists, and numbers of accepted referrals.

Issues around the quality of GP referrals ran through the specialist interviews. As such, we anticipate that the analysis of autism and ADHD referrals using health records might yield further and insights into the level and quality of information required by specialist services.

Finally, it seems likely that GPs in most settings will retain gatekeeping responsibilities for autism and ADHD for the foreseeable future. The findings that some GPs indicated using lay sources such as Google or Wikipedia. As such, we recommended that future work further explores the modes of professional and lay information used by GPs to inform their clinical decision making.

For peer review only - http://bmjopen.bmj.com/site/about/guidelines.xhtml

a. Contributorship statement

BC, MW and RD contributed to the conceptualisation and design of the study. BC applied for governance and ethical approvals, collected the data. BC conducted the initial coding and RD, AM and MW contributed to analysis of the data. Each author offered interpretations of the findings. The final set of themes were agreed by each of the authors. BC wrote the first draft of the manuscript. MW, AM and MW provided critical feedback and suggestions on subsequent drafts. All authors contributed to and approved the final manuscript.

b. Competing interests

None to Declare

c. Funding

The authors wish to thank NIHR School for Primary Care Research [RG94577] for their support for work on this paper. This research was also funded in whole, or in part, by the Wellcome Trust [WT103343MA]. For the purpose of open access, the author has applied a CC BY public copyright licence to any Author Accepted Manuscript version arising from this submission.' We would also like to the CRN for help with recruitment. Warm thanks are extended to authors Prof Marinus van IJzendoorn for feedback on a draft of the manuscript. The views expressed are those of the authors and not necessarily those of the CRN, Wellcome, NHS, the NIHR or the Department of Health.

d. Data sharing statement

Although all participants were reminded not to disclose any personally identifiable information about patients or families, the transcripts do include reflections on routine clinical work and service arrangements. Thus, to further safeguard the privacy or the participants and those involved in their services, we cannot make the transcripts available. Please contact the authors for further details on the data.

For peer review only

References

1. Forrest CB. Primary care in the United States: primary care gatekeeping and referrals: effective filter or failed experiment? BMJ. 2003;326(7391):692-5: 10.1136/bmj.326.7391.692
2. Franks P, Clancy CM, Nutting PA. Gatekeeping revisited—protecting patients from overtreatment. New England Journal of Medicine. 1992;327(6): 10.1056/nejm199208063270613
3. Vedsted P, Olesen F. Are the serious problems in cancer survival partly rooted in gatekeeper principles? An ecologic study. Br J Gen Pract. 2011;61(589):e508-12: 10.3399/bjgp11X588484
4. Sripa P, Hayhoe B, Garg P, Majeed A, Greenfield G. Impact of GP gatekeeping on quality of care, and health outcomes, use, and expenditure: a systematic review. Br J Gen Pract. 2019;69(682):e294-e303: 10.3399/bjgp19X702209
5. Greenfield G, Foley K, Majeed A. Rethinking primary care's gatekeeper role. BMJ. 2016;354:i4803: 10.1136/bmj.i4803
6. Tatlow-Golden M, Prihodova L, Gavin B, Cullen W, McNicholas F. What do general practitioners know about ADHD? Attitudes and knowledge among first-contact gatekeepers: systematic narrative review. BMC family practice. 2016;17(1):129: 10.1186/s12875-016-0516-x
7. Unigwe S, Buckley C, Crane L, Kenny L, Remington A, Pellicano E. GPs' confidence in caring for their patients on the autism spectrum: an online self-report study. Br J Gen Pract. 2017;67(659):e445-e52: 10.3399/bjgp17x690449
8. Kirby A, Davies R, Bryant A. Do teachers know more about specific learning difficulties than general practitioners? British Journal of Special Education. 2005;32(3):122-6: 10.1111/j.0952-3383.2005.00384.x
9. Garg P, Lillystone D, Dossetor D, Kefford C, Chong S. An exploratory survey for understanding perceptions, knowledge and educational needs of general practitioners regarding autistic disorders in New South Wales (NSW), Australia. Journal of clinical and diagnostic research: JCDR. 2014;8(7):PC01: 10.7860/jcdr/2014/8243.4527
10. Coughlan B, Duschinsky R, O'Connor M-E, Woolgar M. Identifying and managing care for children with autism spectrum disorders in general practice: A systematic review and narrative synthesis. Health & Social Care in the Community. 2020;28(6):1928-41: 10.1111/hsc.13098
11. DosReis S, Barksdale CL, Sherman A, Maloney K, Charach A. Stigmatizing experiences of parents of children with a new diagnosis of ADHD. Psychiatr Serv. 2010;61(8):811-6: 10.1176/ps.2010.61.8.811
12. Ryan S, Salisbury H. 'You know what boys are like': pre-diagnosis experiences of parents of children with autism spectrum conditions. Br J Gen Pract. 2012;62(598):e378-e83: 10.3399/bjgp12x641500
13. Boshoff K, Gibbs D, Phillips RL, Wiles L, Porter L. A meta-synthesis of how parents of children with autism describe their experience of advocating for their children during the process of diagnosis. Health & Social Care in the Community. 2019;27(4):e143-e57: 10.1111/hsc.12691
14. Kennedy T, Regehr G, Rosenfield J, Roberts SW, Lingard L. Exploring the gap between knowledge and behavior: a qualitative study of clinician action following an educational intervention. Academic Medicine. 2004;79(5):386-93: 10.1097/00001888-200405000-00006
15. Hyman SL, Levy SE, Myers SM. Identification, Evaluation, and Management of Children With Autism Spectrum Disorder. Pediatrics. 2020;145(1): 10.1542/peds.2019-3447

For peer review only - http://bmjopen.bmj.com/site/about/guidelines.xhtml

16. Thomas R, Spragins W, Mazloum G, Cronkhite M, Maru G. Rates of detection of developmental problems at the 18-month well-baby visit by family physicians' using four evidence-based screening tools compared to usual care: a randomized controlled trial. Child: care, health and development. 2016;42(3):382-93: 10.1111/cch.12333
17. Malterud K, Siersma VD, Guassora AD. Sample Size in Qualitative Interview Studies: Guided by Information Power. Qual Health Res. 2016;26(13):1753-60: 10.1177/1049732315617444
18. Gale NK, Heath G, Cameron E, Rashid S, Redwood S. Using the framework method for the analysis of qualitative data in multi-disciplinary health research. BMC Medical Research Methodology. 2013;13(1):117: 10.1186/1471-2288-13-117
19. Shaw K, Wagner I, Eastwood H, Mitchell G. A qualitative study of Australian GPs' attitudes and practices in the diagnosis and management of attention-deficit/hyperactivity disorder (ADHD). Family Practice. 2003;20(2):129-34: 10.1093/fampra/20.2.129

For peer review only

Case Study 1:

Reception received a phone call from patients Linda (33) and Tim (32) regarding an appointment for their son, Robert (6). The family are known to the practice and previously there have been safeguarding concerns and social services have been involved with the family.

In the initial phone call, Linda requested the next available appointment with the GP. They were subsequently booked in for an appointment in two weeks' time. The next day, Tim phoned reception to express his dissatisfaction with the waiting list and requested that they be given priority in the event of a cancellation.

Two weeks later Linda, Tim, and Robert arrived for the appointment. From the outset, Robert appeared distressed (i.e. crying). Linda made numerous attempts to comfort Robert, but he moved away in response to each of her approaches. At one-point Robert kicked out at Linda. There are what look like two distinctive episodes of hand-flapping.

As the consultation progressed, Robert gradually became more comfortable and was very active (e.g. jumping around the room). He moved from one activity to another in quick succession. Robert's eye contact was fleeting, and seemed to have a restricted range of facial expressions. In terms of conversation, Robert spoke in complex sentences, although the subject matter was a little repetitive and mainly around his favourite toy (Shopkins). Tim then took Robert to the waiting room, so Linda could discuss their concerns with the GP.

According to Linda, Robert has few friends in school and teachers are concerned about his academic progress. Additionally, Robert has become increasingly aggressive towards her and recently threw her laptop at a wall. In terms of history, she reports no significant issues with birth or pregnancy. Robert achieved his motor milestones; however, his language development was delayed. Previously, he received speech and language therapy in the community. Robert has an older half sibling, Chris (14) who has a diagnosis of ADHD. When asked about development prior to three years, Linda disclosed that Robert lived with his grandmother beginning when he was 18 months old to just after his third birthday, as Linda and Tim were separated during this period. During the separation, Linda was an inpatient at a local mental health facility.

For peer review only

Standards for Reporting Qualitative Research (SRQR)*

http://www.equator-network.org/reporting-guidelines/srqr/

Title and abstract

	Page/line no(s).
**Title** - Concise description of the nature and topic of the study Identifying the study as qualitative or indicating the approach (e.g., ethnography, grounded theory) or data collection methods (e.g., interview, focus group) is recommended	Page 3/ line 1/2
**Abstract** - Summary of key elements of the study using the abstract format of the intended publication; typically includes background, purpose, methods, results, and conclusions	Page 3/ line 4

Introduction

**Problem formulation** - Description and significance of the problem/phenomenon studied; review of relevant theory and empirical work; problem statement	Page 4/ line 22
**Purpose or research question** - Purpose of the study and specific objectives or questions	Page 5/line 10

Methods

**Qualitative approach and research paradigm** - Qualitative approach (e.g., ethnography, grounded theory, case study, phenomenology, narrative research) and guiding theory if appropriate; identifying the research paradigm (e.g., postpositivist, constructivist/ interpretivist) is also recommended; rationale**	NA
**Researcher characteristics and reflexivity** - Researchers' characteristics that may influence the research, including personal attributes, qualifications/experience, relationship with participants, assumptions, and/or presuppositions; potential or actual interaction between researchers' characteristics and the research questions, approach, methods, results, and/or transferability	Page 6/ line 28
**Context** - Setting/site and salient contextual factors; rationale**	Page 5//6
**Sampling strategy** - How and why research participants, documents, or events were selected; criteria for deciding when no further sampling was necessary (e.g., sampling saturation); rationale**	Page 6 13/14
**Ethical issues pertaining to human subjects** - Documentation of approval by an appropriate ethics review board and participant consent, or explanation for lack thereof; other confidentiality and data security issues	Page 5/ line 23
**Data collection methods** - Types of data collected; details of data collection procedures including (as appropriate) start and stop dates of data collection and analysis, iterative process, triangulation of sources/methods, and modification of procedures in response to evolving study findings; rationale**	Page 5 line 16

For peer review only - http://bmjopen.bmj.com/site/about/guidelines.xhtml

For peer review only

**Data collection instruments and technologies** - Description of instruments (e.g., interview guides, questionnaires) and devices (e.g., audio recorders) used for data collection; if/how the instrument(s) changed over the course of the study	Page 6 line 21
**Units of study** - Number and relevant characteristics of participants, documents, or events included in the study; level of participation (could be reported in results)	Page 7 Table 1
**Data processing** - Methods for processing data prior to and during analysis, including transcription, data entry, data management and security, verification of data integrity, data coding, and anonymization/de-identification of excerpts	Page 7 line 11
**Data analysis** - Process by which inferences, themes, etc., were identified and developed, including the researchers involved in data analysis; usually references a specific paradigm or approach; rationale**	Page 7 line 6
**Techniques to enhance trustworthiness** - Techniques to enhance trustworthiness and credibility of data analysis (e.g., member checking, audit trail, triangulation); rationale**	Page 7

Results/findings

**Synthesis and interpretation** - Main findings (e.g., interpretations, inferences, and themes); might include development of a theory or model, or integration with prior research or theory	Page 8 - 15
**Links to empirical data** - Evidence (e.g., quotes, field notes, text excerpts, photographs) to substantiate analytic findings	Page 8-15

Discussion

**Integration with prior work, implications, transferability, and contribution(s) to the field -** Short summary of main findings; explanation of how findings and conclusions connect to, support, elaborate on, or challenge conclusions of earlier scholarship; discussion of scope of application/generalizability; identification of unique contribution(s) to scholarship in a discipline or field	Page 15 line 7, Page 16 starting line 5
**Limitations** - Trustworthiness and limitations of findings	

Other

**Conflicts of interest** - Potential sources of influence or perceived influence on study conduct and conclusions; how these were managed	Page 2 line 1
**Funding** - Sources of funding and other support; role of funders in data collection, interpretation, and reporting	Page 1 line 16

*The authors created the SRQR by searching the literature to identify guidelines, reporting standards, and critical appraisal criteria for qualitative research; reviewing the reference lists of retrieved sources; and contacting experts to gain feedback. The SRQR aims to improve the transparency of all aspects of qualitative research by providing clear standards for reporting qualitative research.

For peer review only

For peer review only - http://bmjopen.bmj.com/site/about/guidelines.xhtml

**The rationale should briefly discuss the justification for choosing that theory, approach, method, or technique rather than other options available, the assumptions and limitations implicit in those choices, and how those choices influence study conclusions and transferability. As appropriate, the rationale for several items might be discussed together.

Reference:

O'Brien BC, Harris IB, Beckman TJ, Reed DA, Cook DA. **Standards for reporting qualitative research: a synthesis of recommendations.** *Academic Medicine*, Vol. 89, No. 9 / Sept 2014 DOI: 10.1097/ACM.0000000000000388

For peer review only

For peer review only - http://bmjopen.bmj.com/site/about/guidelines.xhtml

BMJ Open

Clinical perspectives on the identification of neurodevelopmental conditions in children and changes in referral pathways: Qualitative interviews.

Journal:	*BMJ Open*
Manuscript ID	bmjopen-2021-049821.R1
Article Type:	Original research
Date Submitted by the Author:	29-Sep-2021
Complete List of Authors:	Coughlan, Barry; University of Cambridge, Department of Public Health and Primary Care<br>Woolgar, Matt ; King's College London Institute of Psychiatry Psychology and Neuroscience<br>Mann, Alissa; University of Bath, Department of Psychology<br>Duschinsky, Robbie; University of Cambridge Primary Care Unit
**Primary Subject Heading**:	Mental health
Secondary Subject Heading:	Mental health
Keywords:	Community child health < PAEDIATRICS, Developmental neurology & neurodisability < PAEDIATRICS, PRIMARY CARE, Child & adolescent psychiatry < PSYCHIATRY, QUALITATIVE RESEARCH

SCHOLARONE™
Manuscripts

I, the Submitting Author has the right to grant and does grant on behalf of all authors of the Work (as defined in the below author licence), an exclusive licence and/or a non-exclusive licence for contributions from authors who are: i) UK Crown employees; ii) where BMJ has agreed a CC-BY licence shall apply, and/or iii) in accordance with the terms applicable for US Federal Government officers or employees acting as part of their official duties; on a worldwide, perpetual, irrevocable, royalty-free basis to BMJ Publishing Group Ltd ("BMJ") its licensees and where the relevant Journal is co-owned by BMJ to the co-owners of the Journal, to publish the Work in this journal and any other BMJ products and to exploit all rights, as set out in our licence.

The Submitting Author accepts and understands that any supply made under these terms is made by BMJ to the Submitting Author unless you are acting as an employee on behalf of your employer or a postgraduate student of an affiliated institution which is paying any applicable article publishing charge ("APC") for Open Access articles. Where the Submitting Author wishes to make the Work available on an Open Access basis (and intends to pay the relevant APC), the terms of reuse of such Open Access shall be governed by a Creative Commons licence – details of these licences and which Creative Commons licence will apply to this Work are set out in our licence referred to above.

Other than as permitted in any relevant BMJ Author's Self Archiving Policies, I confirm this Work has not been accepted for publication elsewhere, is not being considered for publication elsewhere and does not duplicate material already published. I confirm all authors consent to publication of this Work and authorise the granting of this licence.

For peer review only

Clinical perspectives on the identification of neurodevelopmental conditions in children and changes in referral pathways: Qualitative interviews.

Barry Coughlan[1], Matt Woolgar[2], Alissa Mann,[3,] Robbie Duschinsky[1]

[1]Department of Public Health and Primary Care, University of Cambridge, Cambridge, UK

[2] Institute of Psychiatry, Psychology and Neuroscience, King's College London, London, UK

[3]Department of Psychology, University of Bath

Corresponding author: Mr Barry Coughlan (bc471@medschl.cam.ac.uk); Telephone: 00353830797072

B Coughlan is a PhD candidate and Research Associate. ORCID: 0000-0002-1484-6491

M Woolgar DClinPsy and PhD Consultant Clinical Psychologist; matt.woolgar@kcl.ac.uk; ORCID: 0000-0002-3618-0395

A Mann is a BSc Psychology student at the University of Bath; afm46@bath.ac.uk

R Duschinsky Head of the Applied Social Sciences Group, Department of Public Health and Primary Care at the University of Cambridge; rd522@medschl.cam.ac.uk; ORCID: 0000-0003-2023-5328

Conflicts: No conflicts to declare

For peer review only - http://bmjopen.bmj.com/site/about/guidelines.xhtml

Clinical perspectives on the identification of neurodevelopmental conditions in children and changes in referral pathways: Qualitative interviews.

Word Count = 4552

Abstract

Objective: Previous work has raised questions about the role of General Practitioner's (GPs) in the identification of neurodevelopmental conditions such as autism spectrum disorders (autism) and attention deficit hyperactivity disorders (ADHD).. This study aimed to explore how GPs identify these conditions in practice and their perspectives on recent changes to local referral pathways that mean GP referrals are rarely accepted. We also aimed to explore specialists' views on the role of GPs.

Setting: GP practices, local neurodevelopmental services, and specialist services in the UK.

Participants: Semi-structured interviews were conducted with GPs (n=8), specialists in local services (n=7), and professionals at various specialist services around the country (n=10). Interviews were conducted between January and May 2019. A framework approach informed by thematic analysis was used to analyse the data.

Results: GPs drew on various forms of tacit and explicit information including behavioural markers, parental report, prior knowledge of the family, expert and lay resources. Opinions varied between GPs regarding changes to the referral pathway, with some accepting the changes and others describing it as a "disaster". Specialists tended to feel that GPs required more neurodevelopmental training and time to conduct consultations.

Conclusion: This study adds to the literature showing that GPs use an array of information sources when making referral decisions for autism and ADHD. Further work is urgently required to evaluate the impact of reconfiguring neurodevelopmental referral pathways such that GPs have a diminished role in identification.

Keywords: Autism, ADHD, General Practice, Referral pathways

Strengths:

For peer review only - http://bmjopen.bmj.com/site/about/guidelines.xhtml

- This study uses qualitative interviews and a case study approach which shines important light on GP decision-making processes and their perspectives on changes in referral pathways
- The interview schedule was extensively piloted with various professionals prior to data collection and generated rich data
- Data analysis had inductive and deductive elements building from previous review work

Limitations

- GP were recruited through the local Clinical Research Network (CRN) and thus we are not able to establish whether non-CRN GPs have differing perspectives
- This work is not epistemologically or methodological positioned to comment on the effectiveness of the referral pathways.

Introduction

In the UK General Practitioners (hereafter GPs) are one of the main providers of primary healthcare services. Gatekeeping - the act of determining access to specialist care and diagnostic services - is a routine task for GPs. A core goal of the gatekeeping model is to make healthcare accessible while ensuring that service delivery is feasible. Concerns about the effectiveness of GP gatekeeping are longstanding in the primary care literature e.g. [1 2 3]. Recent reviews have suggested that, in general, GP gatekeeping is linked with a better quality of care and lower service utilisation [4]. Yet questions persist about patient satisfaction with the model and the accuracy of gatekeepers in identifying certain conditions e.g. [4]. In the UK, some clinical commission groups have alleviated GPs of their gatekeeping responsibilities for specific clinical populations, including paediatrics and some mental health services [5]. This has been done by shifting gatekeeping duties to professionals in adjacent fields (e.g., health visitors, social care, and education) or introducing direct referral or self-referral models.

The assessment of developmental conditions such as autism spectrum disorders (autism) and attention deficit hyperactivity disorders (ADHD) reflect these broader tensions around the gatekeeping role in primary care. Referral pathways in the UK often require that GPs initiate referrals for children where there is a query of autism or ADHD. Much of the research on autism and ADHD in general practice focuses on GP knowledge and attitudes towards the respective conditions [6-9]. Survey work indicates that GPs have a sound understanding of autism,

For peer review only - http://bmjopen.bmj.com/site/about/guidelines.xhtml

but little confidence responding to the condition [7]. Still, review work on GP knowledge of autism and ADHD have identified some outmoded aetiological theories still receiving endorsement [6 10]. Consequently, calls for training from GPs and researchers alike are recurrent in much of this work.

Remarkably few studies, however, have explored how GPs make these decisions in practice. This is within a context where parents often describe the pathway to diagnosis as challenging [11-13], and reasons for delays in referral are often felt by parents to be unclear. Some insight, however, can be gleaned from a Canadian study by Kennedy, et al. [14] on medical students at the University of Toronto, which explores knowledge-practice discrepancies following educational programmes. In this qualitative study, the authors identified an array of factors including patient motivations, systemic issues, social and clinician factors as explanations for referral decisions. Increased uncertainty and urgency, somewhat predictably, prompted referrals [14]. Indeed, clinical judgment appears to be an essential factor even within contexts where best practice guidance recommends standardised screening for developmental conditions [15]. For instance, a randomised controlled trial of physicians comparing standard assessments versus traditional methods (i.e. clinical judgment and developmental milestones) found higher detection rates in the group using standardised assessments, and yet referral rates did not significantly differ [16]. This finding prompted the authors to conclude that clinical judgment still overrides standardised assessments.

Our study sought to provide an account of the assessment practices some UK-based GPs engage in when identifying autism and ADHD. This research takes place in an English city where changes to the configuration of local pathways mean that referrals from GPs are rarely accepted. Therefore, a subsidiary aim was to explore how GPs experience these changes and also how clinicians in specialist services think about the role of GPs. Although this study takes place in a particular setting, the themes identified here will have relevance to broader national conversations about the organisation of referral pathways and the gatekeeping role of GPs.

Method

The data presented in this study were collected as part of a project exploring assessment practices in health care professionals (n=25). Specifically, we conducted semi-structured interviews with eight GPs and seven healthcare professionals working across a

For peer review only - http://bmjopen.bmj.com/site/about/guidelines.xhtml
For peer review only

neurodevelopmental team, and child and adolescent mental health services (CAMHS) in an English city. Additionally, we interviewed a further ten professionals who were working at various social and neurodevelopmental services, including tier 4 national services, across the UK. Here we report on the part of the study concerned with GPs experiences of identifying autism and ADHD, changes to local referral pathways, and the views of specialists regarding the role of GPs in the neurodevelopmental assessment. This project was approved by the University of Cambridge Psychology Ethics Committee [PRE.2018.019], The Health Research Authority and local NHS research and development teams. All participants provided written informed consent before data collection. Consent was also provided verbally at the end of each interview.

Referral Pathway

The study was conducted in a socioeconomically diverse English city. Here, community and paediatric teams often work together to provide services for children under five years with a suspected developmental condition including autism and ADHD. This referral pathway is configured such that referrals mostly come from preschools and or health visitors. For school-aged children, referrals tend to go through schools unless the child has an established neurodevelopmental condition. In the first instance, most parents are offered support. Should questions remain about the child's development, then an assessment is conducted by the neurodevelopmental team. Community CAMHS, on the other hand, work with children with mental health problems and accept a referral from an array of sources including GPs, allied healthcare professionals, social workers, and education professionals. There are also teams specialising in child safeguarding.

Data collection

The local clinical research network invited GPs to take part. Professionals from CAMHS, social and neurodevelopmental services were recruited using a combination of purposive, convenience, and snowball sampling techniques. BC conducted all interviews either in person or remotely (e.g. via telephone). Face-to-face interviews were conducted in GP practices or clinic rooms. Data were collected between January and May 2019. For further information, see Table 1. Before data collection, we developed a topic guide based on existing literature and experience of the authors. The guide was piloted with three healthcare professionals working in mental health or developmental services. Questions were also discussed with two academic GPs. The final version of the guide was divided into the following sections: professional

For peer review only - http://bmjopen.bmj.com/site/about/guidelines.xhtml

background, routine clinical work, a hypothetical case study, and referral pathways. See supplement (S1) for the hypothetical case study. At the beginning of each interview, participants were asked not to disclose any personally identifiable information about any patients. Questions in the section on routine clinical work were also prefaced with this reminder (see supplement S2 for interview guide).

BC has experience working in a neurodevelopmental service as an assistant psychologist, where he became interested in the interaction between cognate health services. MW is a consultant clinic psychologist and RD is a social scientist. Both RD and MW are interested in assessment practices for social and neurodevelopmental conditions. AM is a placement student with an interest in child development.

We adopted an 'information power' approach to guide recruitment and sample size [17]. This approach spotlights the following considerations for establishing a sample size in qualitative research: study aim; sample specificity; established theory; quality of dialogue; and analysis strategy [17].

Patient and Public Involvement

A general patient and public review panel at a local hospital provided feedback and suggestions on the research materials, including the topic guide. This panel did not necessarily have specific experience or personal contact with ASD or ADHD.

Data analysis

Data were analysed and interpreted using the framework method outlined by Gale, et al. [18]. This method has the advantage of inductive and deductive elements. This allows for ideas from the existing literature to be brought together with data derived from the interviews to develop an analytical framework. This included a recent systematic review on autism in general practice [10] and a review by Tatlow-Golden and colleagues[6] on GPs and ADHD. All interviews were transcribed by BC or a professional transcription service. Transcripts were read three times, and all audio recordings were listened to at least once before the first round of coding. In the initial stages, transcripts were coded using line-by-line coding. All transcripts were coded by BC, and several of the transcripts were also read in full by AM and RD. Regular meetings were held between the authors to discuss the data. All authors approved the final series of themes. Transcripts were coded by hand, and data were organised and grouped using flashcards. Here,

For peer review only - http://bmjopen.bmj.com/site/about/guidelines.xhtml

results pertaining to identification and referral pathways are discussed. Specialist neurodevelopmental assessment practices and differential conceptualisation are explored elsewhere. Prior to submission, participants were each sent the results and offered the opportunity to comment on the findings.

Table 1: Participant and interview characteristics

Participant ID	Gender	Experience (years)	Setting	Interview Length
**PTGP01**	Female	>20 years	Local GP practice	43 mins
**PTGP02**	Male	4 years	Local GP practice	41 mins
**PTGP03**	Female	>20 years	Local GP practice	44 mins
**PTGP04**	Male	>20 years	Local GP practice	64 mins
**PTGP05**	Male	19 years	Local GP practice	29 mins
**PTGP06**	Male	>20 years	Local GP practice	37 mins
**PTGP07**	Male	>20 years	Local GP practice	71 mins
**PTGP08**	Male	14 years	Local GP practice	61 mins
**PTND01**	Male	17 years	Local ND service	66 mins
**PTND02**	Female	>20 years	Local ND service	64 mins
**PTND03**	Female	13 years	Local ND service	58 mins
**PTND04**	Female	> 20 years	Local ND service	64 mins
**PTND05**	Female	14 years	Local CAMHS	69 mins
**PTND06**	Female	13 years	Lifespan Autism Service	65 mins
**PTND07**	Male	3 years	Child autism service	55 mins
**PTND08**	Female	10 years	Tier 4 CAMHS	62 mins
**PTND09**	Female	19 years	Tier 4 CAMHS	53 mins
**PTND10**	Female	10 years	Tier 4 CAMHS	58 mins
**PTND11**	Female	16 years	Tier 4 CAMHS	48 mins
**PTND12**	Male	6 years	Tier 4 CAMHS	54 mins
**PTND13**	Female	>20 years	Tier 4 CAMHS	43 mins
**PTND14**	Male	>20 years	Tier 4 CAMHS	55 mins
**PTND15**	Female	4 years	Tier 4 CAMHS	61 mins
**PTND16**	Male	4 years	Local CAMHS	63 mins
**PTND17**	Female	>20 years	Local ND	65 mins

ND = Neurodevelopmental, CAMHS = Child and Adolescent Mental Health Services

Results

The findings are presented in two sections. The first section focuses on the methods and sources of information the GPs (n=8) used when identifying autism and ADHD in children. The second section discusses material from the entire set of transcripts (n=25) to explore a range of perspectives on changes to the pathway and the role of the GP. A summary of the main themes is presented in Table 2.

For peer review only - http://bmjopen.bmj.com/site/about/guidelines.xhtml

Table 2: Summary and description of the main themes

	Themes	Description
Identification	Explicit Information	This theme describes forms of information which are considered explicit. This includes reference materials, behavioural markers, and parental report.
	Implicit Information	This theme captures forms of information which are less ostensive than material described above but nevertheless contribute to clinical decisions. This includes clinical intuition and prior knowledge of families.
Referral Pathways	Perceptions of the new referral pathway	This theme provides an account of GPs and specialists impressions of the new pathway.
	Specialist views on the role of GPs	This theme describes specialists' views on the role of GPs.
	Information sharing as a barrier and opportunity	This theme describes participant's views on information sharing between services.

Identification

There was some variation regarding the methods and techniques used by GPs to identify autism and ADHD in children. References to a diverse array of forms of information could be seen across the transcripts, including both tacit and explicit sources. These include various clinical or behavioural markers, unstructured behavioural tasks, prior knowledge of the family, and professional networks and personal experience. Nevertheless, the extent to which GPs considered, used, and triangulated this information varied considerably.

Explicit information: An assortment of diagnostic or clinical markers for each condition were described by participants. That is, practitioners often made reference to specific traits they considered features of certain conditions. Yet some GPs expressed uncertainty and hesitancy when asked about particular indicators:

For peer review only

"Early markers? I'd probably have to look it all up, actually…And often I do. When I've got a patient coming in, I just have a sort of screen what the most common symptoms" PTGP02

"There's gonna[sic] be diagnostic criteria for that but don't ask me what they are. There's a big long list of diagnostic criteria, but I kind of think that's more a specialist job to apply the diagnostic criteria in detail before making the diagnosis, but I'd probably spot the warning signs as it were and refer on as appropriate." PTGP06

And indeed, several practitioners described looking up markers using professional sources such as GP Notebook, Clinical Knowledge Summaries or Patient.co.uk as well as some lay sources including Google or Wikipedia to find specific behavioural markers.

In general, however, GPs appeared to agree on the importance of parental report. This is, of course, understandable as parental concerns are an essential component of the formal assessment for many behaviourally diagnosed developmental conditions. While describing past cases, one GP commented:

"Nine-tenths is the story you're given by the parents. Because they are the… as I say to parents, you know your son or daughter better than anybody in the world. So, we have to listen to what they have to say, [and their] ideas, concerns, and expectations" PTGP07

And indeed, the majority of participants expressed similar sentiments. Importantly, however, most GPs indicated that parental report alone was not sufficient grounds for a referral. Instead, it was suggested that such reports should be corroborated with observations of the child. Yet when facing uncertainty, approaches varied. For instance, after reflecting on complex or uncertain cases, one GP remarked:

"Just got to go with what the parents are thinking" PTGP06

However, another GP was especially concerned with the threat of overdiagnosis. For this GP, it was particularly important to triangulate parental concerns, observations of the child, and reports from the child's school. This GP reflected on a case where parents queried a diagnosis of ADHD following conversations with a family friend:

For peer review only - http://bmjopen.bmj.com/site/about/guidelines.xhtml

"Speaking to the friend caused them to say maybe he [the child] has got ADHD. But in actual fact, I really don't think he has, and the last thing you'd want is for this kid to go on unnecessary medication" PTGP04

He went on to explain that after receiving consent from the child's parents to contact the child's school:

"[I] spoke to his teacher and actually this was an example of where the school actually had a really good handle on him. The teacher said he's a lovely kid, but he's essentially feral. He just isn't set up for rules so there was nothing he's doing at school that would make me worried. He's a lovely lad, and you can engage him, and he can concentrate and focus when he wants to" PTGP04

In contrast, however, there was a least one instance where a GP's decision to refer seemed to be based predominantly on parental insistence, rather than clinical observations or judgment:

"[Refers to another family member] seemed to know it all. [Parent] was saying that they thought the child had autism on the basis that [the child is] behind with learning, not reading and writing yet, didn't like social situations…[…]…. And they said that the school didn't think the child had autism. So, I have referred... I mean [the child] seemed normal, sat doing not a lot, but seemed normal." PTGP05

Subsequently, this participant indicated that the chances of the referral being rejected were '100%' due to the configuration of local referral pathways. When this happens, he explained he would urge the parents to go back to the school.

Tacit information: GPs also often drew implicitly from the language of folk psychology regarding typical and atypical child development. Phrases such as 'a little odd', 'just isn't what most children do" or 'clashes with normal expectations' can be found throughout the data. These were often used in reference to a specific marker or behaviour, such as "rituals and behaviours that weren't quite in keeping with a normal child of her age". Here the term tacit knowledge is used broadly to refer to practical or soft knowledge that is not easily quantifiable.

For peer review only - http://bmjopen.bmj.com/site/about/guidelines.xhtml

Clinical intuition was important for deciding between typical and atypical development, but at times, challenging to articulate:

"As a GP you get a subconscious idea of the spectrum of the range with children - from the kid who'll sit there like butter wouldn't melt in their mouth, like a bit oddly so, to the kid who's climbing up your curtains. [And] You get a feel of parental interaction, with 'you stop doing that now I've told you before' to the parent who just watches the child smash your ophthalmoscope'" PTGP04

"I think it's difficult, sometimes, to describe what turns into a kind of sixth sense. Really you get a clue, don't you? And sort of that kind of gut feeling, but it is about the behaviour." PTGP07

Prior knowledge or experience with specific children and families was also crucial for several GPs. When reflecting on cases, it was not uncommon for practitioners to preface conceptualisations with remarks such as 'I've known him since... well antenatally', 'I know the family' or '[Mum/Dad] is also my patient'. This seemed to offer a degree of context and explanation for the child's presentation. For instance, when describing children with a query of a neurodevelopmental condition, some GPs remarked on traits they had seen in other family members or diagnoses of other family members they were aware of.

GPs were also attuned to socio-environmental or parenting factors that might be contributing to the child's presenting symptoms such as discrete participating events, parental separation or conflict. Having this overview of the patient was, for many, one of the core strengths of general practice:

"I suppose this is where Family Medicine really comes into its fore, isn't it? Because they're [both child and parents] usually, not always, but usually all our patients. So, sometimes we have this interesting dilemma about whom is the patient." PTGP07

Yet this expertise, some felt, was not always appreciated by colleagues in specialist services. When reflecting on the experience of having referrals rejected, one GP remarked:

For peer review only - http://bmjopen.bmj.com/site/about/guidelines.xhtml

“I sometimes wonder whether they [specialist assessment service] actually consider the family factors that we know of that we write in out letters” PTGP03

Perceptions of the new referral pathway

Most of the GPs were aware of the changes to the assessment pathway that meant referrals for neurodevelopmental assessment typically come through schools or health visitors. However, it was unclear whether two GPs were aware of these changes. Three stances to these changes were identified in the data: accepting, ambivalent, and critical. Practitioners who were more accepting of the changes trended to reason that schools are better positioned to identify such developmental conditions:

“Well, you see I think community paediatrics probably has a point. Because small child gets brought in to see the doctor and they’re looking around looking reasonably normal but what do I know. Whereas the school and other people that interact with the child over a long period of time are in a better position to make an assessment than me.” PTGP05

“They’ll [schools] be better at recognising it than me, so I’m happy, doesn’t matter where the referral comes from, as long as it happens in a timely way it doesn’t have to come from a GP.” PTGP06

One participant had a more ambivalent attitude. For this participant, there was an acknowledgement that schools are often well-placed to identify atypical development. Still, they maintained that limiting the ability of GPs to refer put them in a challenging position:

“Difficult. Because you can see the logic in that, actually there’s so much more to this than having a name put to your child’s odd behaviour very few of these children will benefit from something medical…[…]… the problem comes really when a parent comes in and says I’ve been to the teacher three times, and the teacher says they think he’s fine and if you’re really worried you can go and see your GP. Because you’ve no idea did the teacher really say that.” PTGP04

While one GP was critical of the pathway:

For peer review only - http://bmjopen.bmj.com/site/about/guidelines.xhtml

"Just a disaster, just a road crash really - trying to get children seen with developmental or behavioural problems is increasingly difficult, and in fact, for many patients, we end up having to go if they're school age we end up having to go through school…[…]... And that's a real nightmare for me because it means I'm having to delegate that to a third party who is not actually a health service" PTGP07

This GP felt that the pathway was also a threat to professional status and identity, reflecting a devaluation of primary care.

Professionals in the neurodevelopmental services tended to view the changes as positive. Professionals in the neurodevelopmental team reported on the impact these changes have had on service-level pressures, including waiting times for assessment:

"We've got the shortest waiting times for assessment for autism and ADHD. Less than eighteen weeks, whereas they were eighteen months to two to three years [before]" PTND01

Specialist views on the role of GPs

When asked directly whether GPs had a role in identifying developmental conditions, most specialists indicated that there was indeed a role for GPs. Yet this was often couched with an array of caveats about professional and organisational barriers to identification. The most common barrier, according to the specialists, was the duration of primary care consultations and a lack of training or knowledge about neurodevelopmental conditions:

"They need to be given more time to do it properly and more training. They get very little training at all really but if they got proper training and given a bit more time. Even fifteen-twenty minutes, but at the moment all they could do is to at least know the NICE guidance and know what are the signs and symptoms and take a detailed history and follow the local pathway really. Clearly, if we have GPs with a special interest in children, they got better training, and clearly, they have a lot of role to play with the ADHD medication shared care and those kinds of things." PTND01

"At best what they should do is make good referrals to specialist teams. But beyond that, I don't know if it would be useful for people who are under massive strain and pressure and who have like whatever is it eight to ten-minute appointments, I hear that's the average, but I've

For peer review only - http://bmjopen.bmj.com/site/about/guidelines.xhtml

never had any more than six minutes really, so I mean I don't know how you could do anything bar account for the family's request and signpost them to the appropriate teams." PTND07

A lack of training was also framed as problematic by one GP:

"I think also in terms of what we get taught it may change now obviously I trained thirty years ago literally we had no training at all...[…]… We'd all heard of autism but everything I know about neurodevelopmental disorders, not that there's much of it, has been acquired post-grad." PTGP04

References to the time afforded for consultations can also be found throughout the GP interviews. For some, this was felt to be a significant barrier to identification. To circumnavigate some of these challenges, one GP described bringing families back for multiple consultations.

Information sharing as a barrier and opportunity:

Another topic that runs through the data is the importance of informal networks and the issues with sharing information between services. In general, informal networks could be described as internal and external. Internal networks mostly consisted of practice staff, including administrative staff, GP colleagues and nurses. By contrast, external networks consisted of educational professionals and colleagues in secondary care. Due to the reconfiguration of primary care services, health visitors seemed to occupy a position between these two networks:

"We used to have Health Visitors attached to the practice, but they don't exist anymore. I don't know who our Health Visitor is I've never met them." PTGP05

Meanwhile, most GPs acknowledged that nursery staff, primary school teachers and other educational professionals were essential sources of information when thinking about child development. Yet the lack of a linked system for educational and primary care records presented challenges in terms of sharing this information:

"We have occasional contact with schools but not very much. Not often. I'd be unsure about the boundaries and confidentiality and things like that, to be honest." PTGP05

For peer review only - http://bmjopen.bmj.com/site/about/guidelines.xhtml

There also seemed to be a lack of communication between GPs and specialist services:

"It's so difficult because you know you'll write the letter, but you don't know if they'll actually get any help or whether they'll get put on the waiting list or whether someone else will monitor the child. So that's the tricky bit really." PTGP03

"let's say we're querying autism they [neurodevelopmental team] would send the referral back. And say it needs to be referred through the school which is quite doable because quite often they have started with the school. And the school have said have you seen your GP and of course then it looks like passing the parcel." PTGP04

Discussion

Summary

GPs used tacit and explicit forms of information when identifying autism and ADHD in children. These included clinical or behavioural markers, parental report, prior knowledge of the child and family, and professional networks. For most, parental concerns were the chief factor driving referral decisions. However, a few participants described instances where they had sought information from other sources (e.g. schools). Nevertheless, changes to the configuration of local pathways have meant that referrals from GPs for neurodevelopmental assessment are now rarely accepted. GPs had mixed views on these changes. Most specialists agreed that GPs did have a role in identifying neurodevelopmental conditions yet expressed concerns about a perceived lack of training or knowledge and framed time pressures as problematic.

Strengths and Limitations

The current study adds to our understanding of early identification by gleaning the perspectives of GPs and those in specialists' assessment services. From a methodological perspective, the flexible interview guide and the combination of case-based discussions and clinical vignette allowed us to elicit rich narratives about these topics. Further, by analysing discourses of past and hypothetical cases, we were able to explore some of the other forms of knowledge that come into play. Additionally, our study was conducted in a setting where GPs have been, to a large extent, absolved of their gatekeeping responsibilities for identifying autism and ADHD in children. Therefore, the current study presents a unique opportunity to explore how GPs

For peer review only - http://bmjopen.bmj.com/site/about/guidelines.xhtml

experience having a reduced role for a specific patient group and thus adds to national conversations about the nature and future of general practice. That said, it is essential to consider whether the findings about identification are transferable to other contexts. Regarding identification, given that specialists espoused similar issues with referrals in different settings, it seems unlikely that the methods and techniques used by GPs in this area were atypical. As recruitment of GPs was completed through the local CRN, it is not possible to determine how many GPs decided not to take part in the study. This might raise other concerns about the representativeness of the GP sample. However, as the analysis illustrates, there was considerable diversity in the views and opinions expressed by the GPs. Finally, another limitation of this study is that, although data were discussed at regular meetings between the research group, BC conducted and coded the analysis. As per the method, BC has previously worked in a neurodevelopmental service. To address possible issues with research bias, BC wrote reflections throughout the process and engaged in peer and academic supervision.

Comparison with the literature

Most studies about GP knowledge of autism and ADHD have focused on explicit knowledge of clinical markers [6 7 9 19]. Yet, as others have shown, clinical judgment is core to referral decisions [14 16]. Naturally, knowledge of clinical markers is important for identifying these conditions. For ADHD, some co-developed training tools are showing promise [20]. Still, an overemphasis on this form of knowledge risks driving attention away from the other sources GPs draw on, including prior experience with the child or family. Our study, therefore, adds to the understanding of identification by tracing out the various forms of explicit and tacit material which GPs draw upon when determining whether a child requires formal assessment.

Several studies have identified that GPs frequently report having little training in autism e.g. [7] and ADHD [6]. It follows that more training is needed. Our data lend some support to these findings and broadly speaking, we agree with these calls for more training. The 'lack of training' thread runs throughout the primary care literature. However, a degree of caution is warranted, as framing the problem as one of 'a lack of training' risks a) flattening the conceptual complexity associated with identifying these conditions b) silencing the host of organisational shortcomings that make referral decisions challenging, and c) camouflaging alternate solutions such as the integration of health, educational, justice or social care records or changing pathways.

For peer review only - http://bmjopen.bmj.com/site/about/guidelines.xhtml

Implications for Research and Practice

Elsewhere, questions have been raised about GP gatekeeping [1-3]. As such, zooming in on a particular pathway means that we were able to explore in detail how those on the ground experienced changes to GP gatekeeping. It might be envisaged that GPs would welcome changes that reduce some of the pressure on them. Yet GPs in this study expressed mixed views. In contrast, specialists tended to view the changes positively and credit these changes with preventing saturation of the service. Our research is not positioned to explore the impact that these changes have on service delivery. We recommend that future work explores how such changes impact patient satisfaction, waiting lists, and numbers of accepted referrals.

Issues around the quality of GP referrals ran through the specialist interviews. As such, we anticipate that the analysis of autism and ADHD referrals using health records might yield further insights into the level and quality of information required by specialist services.

Finally, it seems likely that GPs in most settings will retain gatekeeping responsibilities for autism and ADHD for the foreseeable future. The findings indicate that some GPs used lay sources such as Google or Wikipedia. As such, we recommended that future work further explores the modes of professional and lay information used by GPs to inform their clinical decision making. In particular, we would welcome research that explores whether the forms of information used by GPs has an impact on referral decisions and on referral acceptance.

For peer review only

a. Contributorship statement

BC, MW and RD contributed to the conceptualisation and design of the study. BC applied for governance and ethical approvals, and collected the data. BC conducted the initial coding while RD, AM and MW contributed to analysis of the data. Each author offered interpretations of the findings. The final set of themes were agreed by each of the authors. BC wrote the first draft of the manuscript. MW, AM and MW provided critical feedback and suggestions on subsequent drafts. All authors contributed to and approved the final manuscript.

b. Competing interests

None to Declare

c. Funding

The authors wish to thank NIHR School for Primary Care Research [RG94577] for their support for work on this paper. This research was also funded in whole, or in part, by the Wellcome Trust [WT103343MA]. For the purpose of open access, the author has applied a CC BY public copyright licence to any Author Accepted Manuscript version arising from this submission.' We would also like to the CRN for help with recruitment. Warm thanks are extended to authors Prof Marinus van IJzendoorn for feedback on a draft of the manuscript. The views expressed are those of the authors and not necessarily those of the CRN, Wellcome, NHS, the NIHR or the Department of Health.

d. Data sharing statement

Although all participants were reminded not to disclose any personally identifiable information about patients or families, the transcripts do include reflections on routine clinical work and service arrangements. Thus, to further safeguard the privacy or the participants and those involved in their services, we cannot make the transcripts available. Please contact the authors for further details on the data.

Ethics Statement

For peer review only - http://bmjopen.bmj.com/site/about/guidelines.xhtml

This project was approved by the University of Cambridge Psychology Ethics Committee [PRE.2018.019].

For peer review only

For peer review only - http://bmjopen.bmj.com/site/about/guidelines.xhtml

References
1. Forrest CB. Primary care in the United States: primary care gatekeeping and referrals: effective filter or failed experiment? *BMJ* 2003;326(7391):692-5. doi: 10.1136/bmj.326.7391.692 [published Online First: 2003/03/29]
2. Franks P, Clancy CM, Nutting PA. Gatekeeping revisited—protecting patients from overtreatment. *New England Journal of Medicine* 1992;327(6) doi: 10.1056/nejm199208063270613
3. Vedsted P, Olesen F. Are the serious problems in cancer survival partly rooted in gatekeeper principles? An ecologic study. *Br J Gen Pract* 2011;61(589):e508-12. doi: 10.3399/bjgp11X588484 [published Online First: 2011/08/02]
4. Sripa P, Hayhoe B, Garg P, et al. Impact of GP gatekeeping on quality of care, and health outcomes, use, and expenditure: a systematic review. *Br J Gen Pract* 2019;69(682):e294-e303. doi: 10.3399/bjgp19X702209 [published Online First: 2019/03/27]
5. Greenfield G, Foley K, Majeed A. Rethinking primary care's gatekeeper role. *BMJ* 2016;354:i4803. doi: 10.1136/bmj.i4803 [published Online First: 2016/09/25]
6. Tatlow-Golden M, Prihodova L, Gavin B, et al. What do general practitioners know about ADHD? Attitudes and knowledge among first-contact gatekeepers: systematic narrative review. *BMC family practice* 2016;17(1):129. doi: 10.1186/s12875-016-0516-x
7. Unigwe S, Buckley C, Crane L, et al. GPs' confidence in caring for their patients on the autism spectrum: an online self-report study. *Br J Gen Pract* 2017;67(659):e445-e52. doi: 10.3399/bjgp17x690449
8. Kirby A, Davies R, Bryant A. Do teachers know more about specific learning difficulties than general practitioners? *British Journal of Special Education* 2005;32(3):122-26. doi: 10.1111/j.0952-3383.2005.00384.x
9. Garg P, Lillystone D, Dossetor D, et al. An exploratory survey for understanding perceptions, knowledge and educational needs of general practitioners regarding autistic disorders in New South Wales (NSW), Australia. *Journal of clinical and diagnostic research: JCDR* 2014;8(7):PC01. doi: 10.7860/jcdr/2014/8243.4527
10. Coughlan B, Duschinsky R, O'Connor M-E, et al. Identifying and managing care for children with autism spectrum disorders in general practice: A systematic review and narrative synthesis. *Health & Social Care in the Community* 2020;28(6):1928-41. doi: 10.1111/hsc.13098
11. DosReis S, Barksdale CL, Sherman A, et al. Stigmatizing experiences of parents of children with a new diagnosis of ADHD. *Psychiatr Serv* 2010;61(8):811-6. doi: 10.1176/ps.2010.61.8.811 [published Online First: 2010/08/03]
12. Ryan S, Salisbury H. 'You know what boys are like': pre-diagnosis experiences of parents of children with autism spectrum conditions. *Br J Gen Pract* 2012;62(598):e378-e83. doi: 10.3399/bjgp12x641500
13. Boshoff K, Gibbs D, Phillips RL, et al. A meta-synthesis of how parents of children with autism describe their experience of advocating for their children during the process of diagnosis. *Health & Social Care in the Community* 2019;27(4):e143-e57. doi: 10.1111/hsc.12691
14. Kennedy T, Regehr G, Rosenfield J, et al. Exploring the gap between knowledge and behavior: a qualitative study of clinician action following an educational intervention. *Academic Medicine* 2004;79(5):386-93. doi: 10.1097/00001888-200405000-00006
15. Hyman SL, Levy SE, Myers SM. Identification, Evaluation, and Management of Children With Autism Spectrum Disorder. *Pediatrics* 2020;145(1) doi: 10.1542/peds.2019-3447 [published Online First: 2019/12/18]
16. Thomas R, Spragins W, Mazloum G, et al. Rates of detection of developmental problems at the 18-month well-baby visit by family physicians' using four evidence-based screening tools compared to usual care: a randomized controlled trial. *Child: care, health and development* 2016;42(3):382-93. doi: 10.1111/cch.12333
17. Malterud K, Siersma VD, Guassora AD. Sample Size in Qualitative Interview Studies: Guided by Information Power. *Qual Health Res* 2016;26(13):1753-60. doi: 10.1177/1049732315617444 [published Online First: 2015/11/29]
18. Gale NK, Heath G, Cameron E, et al. Using the framework method for the analysis of qualitative data in multi-disciplinary health research. *BMC Medical Research Methodology* 2013;13(1):117. doi: 10.1186/1471-2288-13-117

For peer review only - http://bmjopen.bmj.com/site/about/guidelines.xhtml

19. Shaw K, Wagner I, Eastwood H, et al. A qualitative study of Australian GPs' attitudes and practices in the diagnosis and management of attention-deficit/hyperactivity disorder (ADHD). *Family Practice* 2003;20(2):129-34. doi: 10.1093/fampra/20.2.129
20. French B, Daley D, Perez Vallejos E, et al. Development and evaluation of an online education tool on attention deficit hyperactivity disorder for general practitioners: the important contribution of co-production. *BMC Family Practice* 2020;21(1):224. doi: 10.1186/s12875-020-01289-5

For peer review only

Case Study 1:
Reception received a phone call from patients Linda (33) and Tim (32) regarding an appointment for their son, Robert (6). The family are known to the practice and previously there have been safeguarding concerns and social services have been involved with the family.

In the initial phone call, Linda requested the next available appointment with the GP. They were subsequently booked in for an appointment in two weeks' time. The next day, Tim phoned reception to express his dissatisfaction with the waiting list and requested that they be given priority in the event of a cancellation.

Two weeks later Linda, Tim, and Robert arrived for the appointment. From the outset, Robert appeared distressed (i.e. crying). Linda made numerous attempts to comfort Robert, but he moved away in response to each of her approaches. At one-point Robert kicked out at Linda. There are what look like two distinctive episodes of hand-flapping.

As the consultation progressed, Robert gradually became more comfortable and was very active (e.g. jumping around the room). He moved from one activity to another in quick succession. Robert's eye contact was fleeting, and seemed to have a restricted range of facial expressions. In terms of conversation, Robert spoke in complex sentences, although the subject matter was a little repetitive and mainly around his favourite toy (Shopkins). Tim then took Robert to the waiting room, so Linda could discuss their concerns with the GP.

According to Linda, Robert has few friends in school and teachers are concerned about his academic progress. Additionally, Robert has become increasingly aggressive towards her and recently threw her laptop at a wall. In terms of history, she reports no significant issues with birth or pregnancy. Robert achieved his motor milestones; however, his language development was delayed. Previously, he received speech and language therapy in the community. Robert has an older half sibling, Chris (14) who has a diagnosis of ADHD. When asked about development prior to three years, Linda disclosed that Robert lived with his grandmother beginning when he was 18 months old to just after his third birthday, as Linda and Tim were separated during this period. During the separation, Linda was an inpatient at a local mental health facility.

For peer review only

Supplement 2. Full study interview guide

Introduction:

I would like to ask you some questions about your training and background, your routine clinical activities, and your experiences and views on the diagnostic process for developmental conditions in children. I would like to remind you not to disclose any personally identifiable information about any individuals, child or families you work with during the interview.

Background
Can you give me a brief overview of your current position?
Probe for experience related to child development

How long have you been working as a (GP, psychologist, psychiatrist etc)?
Probe for length of time working with children and families

Where and when did you do your clinical training?
Probe for General Practitioner (GP) training vs medical training

Clinical work

Without revealing any personally identifiable information, can you tell me about a case where you conceptualised the child's features as relating to autism?
Probe action steps
Probe specific difficulties with working with this child
Probe for informal assessment (e.g. familiarity with things in the past)
Probe assessments (I know X is saturated with screening tools/assessments; observations)
Probe for level of clarity (how certain or uncertain were you about X)
Repeat for ADHD and attachment related

If not clear, can you give me an overview of your involvement in relation to identification and assessment of developmental (or behavioural) difficulties?
Probe for assessment tools, observations and focus on developmental histories, team-based approach etc.
If GP, probe for screening tools.

Can you think of a specific instance where there was uncertainty regarding the nature of a child's difficulties/diagnosis?
Probe for description
Probe for action steps

Are there other factors that contributed/routinely contribute to the decision you made (e.g. system factors, availability of supports and services)?
Have you ever been involved with overturning or changing a child's diagnosis?

Case Study Questions

For peer review only

Questions for participants with Case Study 1,

What are your initial thoughts on the case?
What questions would you ask A) Robert B) Parents (Tim & Linda)
What would you advise as the next steps?
If the participant deems onward referral is necessary
what information would you put in the referral letter?
Why did X resonate with you?
How easy have you found it to get external help

What did you find yourself drawing on to make sense of Roberts presenting difficulties? (i.e. other similar cases, diagnostic criteria, theory etc)

If the child had a private diagnosis of autism, would that influence how you think about the case?

If you were unsure about the nature of the child's difficulties, are there other professionals you would consult with?

Questions for participants with Case study 2.

What are your initial thoughts on the case?
Probe for possible diagnosis, working clinical hypotheses, formulation

If the participant states a specific diagnosis/conceptualisation
What features of the case helped you to arrive at that conclusion?
Are there other conditions you considered?
Probe, if so what?
Probe how the participant differentiated

If the participant indicates that it could be multiple conditions
You mentioned that it could be X or Y, how would you differentiate?
What assessments/sources of information would be useful in helping you to reach your decision?

If the participant indicates there isn't enough information to make the decision
What information would help you make a decision regarding the nature of the child's difficulties?
Are there specific assessments/ theories you would use?

In your view, is there need for further assessment?
If Yes, what are the next steps in terms of assessment?

Probe for risk assessment
Probe cognitive assessment
Probe sources of information

Are there any frameworks/ theories you used to understand the nature of the child's presenting difficulties?

For peer review only

Probe for specific models/ theories
Probe for influential book/text/ or talks

If the child had a private diagnosis of autism, would that influence how you think about the case?

If you were unsure about the nature of the child's difficulties, are there other professionals you would consult with?
Referral Pathways Information

In your experience, what do you consider the early markers of autism?
Probe for symptoms, features, predisposing events, comorbid conditions, family history
**repeat for ADHD, Attachment related difficulties.

Rotate order each interview

Questions for Primary Care Clinicians
Have you ever referred a child to a CAMHS or relevant assessment service as you suspected the child may a developmental condition or behavioural difficulty?
If yes, what information did you include in your referral letter?
Have you ever had a referral of this nature not accepted in the first instance?
If yes, why was the referral not accepted?
On a scale of 1-10, how easy have you found it to get formal assessment for a child you think refer a child who you think may have autism?
Repeat for attachment problems
Repeat for ADHD
Have you experienced any challenges to referring a child who you think may have autism?
Repeat for attachment problems
Repeat for ADHD
What, if anything could be done to make the referral pathway, clearer?
On a scale of 1-10, how confident do you feel that you will be able to get the appropriate a) assessment b) support for a child with autism, adhd, attachment problems.

Questions for non-primary care clinicians
Do you receive many referrals to your service from GPs?
If yes, how would you describe the quality of these referrals?
Probe what's in a good/less good referral
If no, where do you get the majority of referrals from
How would you describe the quality of these referrals?
What information would constitute a sufficient referral (i.e. allows you to make your decision regarding acceptance for subsequent assessment/support)
What information could be collected by a GP to assist with the decision to accept or reject a referral to your service?
How do you think children and families experience the journey from primary care to your service?
Thinking about conditions with overlapping features, do you see value in differentiating autism from attachment-related conditions?
Do you see a meaningful distinction between the attachment disorders (RAD and Disinhibited) and attachment-related difficulties?

For peer review only

Similarly, when considering a diagnosis of autism and adhd, what value do you see in diagnosing both or trying to differentiate?
Finally, what in you view is the value in differentiating ADHD from attachment-related conditions?

For peer review only

For peer review only

Standards for Reporting Qualitative Research (SRQR)*

http://www.equator-network.org/reporting-guidelines/srqr/

Title and abstract

	Page/line no(s).
**Title** - Concise description of the nature and topic of the study Identifying the study as qualitative or indicating the approach (e.g., ethnography, grounded theory) or data collection methods (e.g., interview, focus group) is recommended	Page 3/ line 1/2
**Abstract** - Summary of key elements of the study using the abstract format of the intended publication; typically includes background, purpose, methods, results, and conclusions	Page 3/ line 4

Introduction

**Problem formulation** - Description and significance of the problem/phenomenon studied; review of relevant theory and empirical work; problem statement	Page 4/ line 22
**Purpose or research question** - Purpose of the study and specific objectives or questions	Page 5/line 10

Methods

**Qualitative approach and research paradigm** - Qualitative approach (e.g., ethnography, grounded theory, case study, phenomenology, narrative research) and guiding theory if appropriate; identifying the research paradigm (e.g., postpositivist, constructivist/ interpretivist) is also recommended; rationale**	NA
**Researcher characteristics and reflexivity** - Researchers' characteristics that may influence the research, including personal attributes, qualifications/experience, relationship with participants, assumptions, and/or presuppositions; potential or actual interaction between researchers' characteristics and the research questions, approach, methods, results, and/or transferability	Page 6/ line 28
**Context** - Setting/site and salient contextual factors; rationale**	Page 5//6
**Sampling strategy** - How and why research participants, documents, or events were selected; criteria for deciding when no further sampling was necessary (e.g., sampling saturation); rationale**	Page 6 13/14
**Ethical issues pertaining to human subjects** - Documentation of approval by an appropriate ethics review board and participant consent, or explanation for lack thereof; other confidentiality and data security issues	Page 5/ line 23
**Data collection methods** - Types of data collected; details of data collection procedures including (as appropriate) start and stop dates of data collection and analysis, iterative process, triangulation of sources/methods, and modification of procedures in response to evolving study findings; rationale**	Page 5 line 16

For peer review only

**Data collection instruments and technologies** - Description of instruments (e.g., interview guides, questionnaires) and devices (e.g., audio recorders) used for data collection; if/how the instrument(s) changed over the course of the study	Page 6 line 21
**Units of study** - Number and relevant characteristics of participants, documents, or events included in the study; level of participation (could be reported in results)	Page 7 Table 1
**Data processing** - Methods for processing data prior to and during analysis, including transcription, data entry, data management and security, verification of data integrity, data coding, and anonymization/de-identification of excerpts	Page 7 line 11
**Data analysis** - Process by which inferences, themes, etc., were identified and developed, including the researchers involved in data analysis; usually references a specific paradigm or approach; rationale**	Page 7 line 6
**Techniques to enhance trustworthiness** - Techniques to enhance trustworthiness and credibility of data analysis (e.g., member checking, audit trail, triangulation); rationale**	Page 7

Results/findings

**Synthesis and interpretation** - Main findings (e.g., interpretations, inferences, and themes); might include development of a theory or model, or integration with prior research or theory	Page 8 - 15
**Links to empirical data** - Evidence (e.g., quotes, field notes, text excerpts, photographs) to substantiate analytic findings	Page 8-15

Discussion

**Integration with prior work, implications, transferability, and contribution(s) to the field** - Short summary of main findings; explanation of how findings and conclusions connect to, support, elaborate on, or challenge conclusions of earlier scholarship; discussion of scope of application/generalizability; identification of unique contribution(s) to scholarship in a discipline or field	Page 15 line 7, Page 16 starting line 5
**Limitations** - Trustworthiness and limitations of findings	

Other

**Conflicts of interest** - Potential sources of influence or perceived influence on study conduct and conclusions; how these were managed	Page 2 line 1
**Funding** - Sources of funding and other support; role of funders in data collection, interpretation, and reporting	Page 1 line 16

*The authors created the SRQR by searching the literature to identify guidelines, reporting standards, and critical appraisal criteria for qualitative research; reviewing the reference lists of retrieved sources; and contacting experts to gain feedback. The SRQR aims to improve the transparency of all aspects of qualitative research by providing clear standards for reporting qualitative research.

For peer review only

For peer review only - http://bmjopen.bmj.com/site/about/guidelines.xhtml

**The rationale should briefly discuss the justification for choosing that theory, approach, method, or technique rather than other options available, the assumptions and limitations implicit in those choices, and how those choices influence study conclusions and transferability. As appropriate, the rationale for several items might be discussed together.

Reference:

O'Brien BC, Harris IB, Beckman TJ, Reed DA, Cook DA. **Standards for reporting qualitative research: a synthesis of recommendations.** *Academic Medicine*, Vol. 89, No. 9 / Sept 2014 DOI: 10.1097/ACM.0000000000000388

For peer review only

For peer review only - http://bmjopen.bmj.com/site/about/guidelines.xhtml

BMJ Open

Clinical perspectives on the identification of neurodevelopmental conditions in children and changes in referral pathways: Qualitative interviews.

Journal:	*BMJ Open*
Manuscript ID	bmjopen-2021-049821.R2
Article Type:	Original research
Date Submitted by the Author:	04-Mar-2022
Complete List of Authors:	Coughlan, Barry; University of Cambridge, Department of Public Health and Primary Care<br>Woolgar, Matt ; King's College London Institute of Psychiatry Psychology and Neuroscience<br>Mann, Alissa; University of Bath, Department of Psychology<br>Duschinsky, Robbie; University of Cambridge Primary Care Unit
**Primary Subject Heading**:	Mental health
Secondary Subject Heading:	Mental health
Keywords:	Community child health < PAEDIATRICS, Developmental neurology & neurodisability < PAEDIATRICS, PRIMARY CARE, Child & adolescent psychiatry < PSYCHIATRY, QUALITATIVE RESEARCH

SCHOLARONE™
Manuscripts

I, the Submitting Author has the right to grant and does grant on behalf of all authors of the Work (as defined in the below author licence), an exclusive licence and/or a non-exclusive licence for contributions from authors who are: i) UK Crown employees; ii) where BMJ has agreed a CC-BY licence shall apply, and/or iii) in accordance with the terms applicable for US Federal Government officers or employees acting as part of their official duties; on a worldwide, perpetual, irrevocable, royalty-free basis to BMJ Publishing Group Ltd ("BMJ") its licensees and where the relevant Journal is co-owned by BMJ to the co-owners of the Journal, to publish the Work in this journal and any other BMJ products and to exploit all rights, as set out in our licence.

The Submitting Author accepts and understands that any supply made under these terms is made by BMJ to the Submitting Author unless you are acting as an employee on behalf of your employer or a postgraduate student of an affiliated institution which is paying any applicable article publishing charge ("APC") for Open Access articles. Where the Submitting Author wishes to make the Work available on an Open Access basis (and intends to pay the relevant APC), the terms of reuse of such Open Access shall be governed by a Creative Commons licence – details of these licences and which Creative Commons licence will apply to this Work are set out in our licence referred to above.

Other than as permitted in any relevant BMJ Author's Self Archiving Policies, I confirm this Work has not been accepted for publication elsewhere, is not being considered for publication elsewhere and does not duplicate material already published. I confirm all authors consent to publication of this Work and authorise the granting of this licence.

For peer review only

Clinical perspectives on the identification of neurodevelopmental conditions in children and changes in referral pathways: Qualitative interviews.

Barry Coughlan[1], Matt Woolgar[2], Alissa Mann,[3,] Robbie Duschinsky[1]

[1]Department of Public Health and Primary Care, University of Cambridge, Cambridge, UK

[2] Institute of Psychiatry, Psychology and Neuroscience, King's College London, London, UK

[3]Department of Psychology, University of Bath

Corresponding author: Dr Barry Coughlan (bc471@medschl.cam.ac.uk); Telephone: 00353830797072

B Coughlan Postdoctoral Research Associate and Deputy Head of the Applied Social Sciences Group Department of Public Health and Primary Care at the University of Cambridge; ORCID: 0000-0002-1484-6491

M Woolgar DClinPsy and PhD Consultant Clinical Psychologist; matt.woolgar@kcl.ac.uk; ORCID: 0000-0002-3618-0395

A Mann is a BSc Psychology student at the University of Bath; afm46@bath.ac.uk

R Duschinsky Head of the Applied Social Sciences Group, Department of Public Health and Primary Care at the University of Cambridge; rd522@medschl.cam.ac.uk; ORCID: 0000-0003-2023-5328

Conflicts: No conflicts to declare

For peer review only

Clinical perspectives on the identification of neurodevelopmental conditions in children and changes in referral pathways: Qualitative interviews.

Word Count = 4552

Abstract

Objective: Previous work has raised questions about the role of General Practitioners (GPs) in the identification of neurodevelopmental conditions such as autism spectrum disorders (autism) and attention deficit hyperactivity disorders (ADHD). This study aimed to explore how GPs identify these conditions in practice and their perspectives on recent changes to local referral pathways that mean referrals to the neurodevelopmental team come through educational professionals and health visitors, rather than GPs. This study also aimed to explore child and adolescent mental health services (CAMHS) specialist's perspectives on the role of GPs.

Setting: GP practices, local neurodevelopmental services, and specialist CAMHS services in the UK.

Participants: Semi-structured interviews were conducted with GPs (n=8), specialists in local CAMHS (n=7), and professionals at national CAMHS services around the country (n=10). Interviews were conducted between January and May 2019. A framework approach informed by thematic analysis was used to analyse the data.

Results: GPs drew on various forms of tacit and explicit information including behavioural markers, parental report, prior knowledge of the family, expert and lay resources. Opinions varied between GPs regarding changes to the referral pathway, with some accepting the changes and others describing it as a "disaster". CAMHs specialists tended to feel that GPs required more neurodevelopmental training and time to conduct consultations.

Conclusion: This study adds to the literature showing that GPs use an array of information sources when making referral decisions for autism and ADHD. Further work is urgently required to evaluate the impact of reconfiguring neurodevelopmental referral pathways such that GPs have a diminished role in identification.

Keywords: Autism, ADHD, General Practice, Referral pathways

For peer review only - http://bmjopen.bmj.com/site/about/guidelines.xhtml

Strengths:

- This study uses qualitative interviews and a hypothetical case study approach; by doing so shines important light on GP decision-making processes and their perspectives on changes to referral pathways
- The interview schedule was extensively piloted with various professionals prior to data collection and generated rich data
- Data analysis had inductive and deductive elements building from previous review work

Limitations

- GPs were recruited through the local Clinical Research Network (CRN). Therefore, we did not capture the practices and perspectives of GPs not actively involved in research through the CRN.
- This work is not epistemologically, or methodological positioned to comment on the effectiveness of the referral pathways.

Introduction

In the UK, General Practitioners (hereafter GPs) are one of the main providers of primary healthcare services. Gatekeeping - the act of determining access to specialist care and diagnostic services - is a routine task for GPs. A core goal of the gatekeeping model is to make healthcare accessible while ensuring that service delivery is feasible. Concerns about the effectiveness of GP gatekeeping are longstanding in the primary care literature, e.g. [1 2 3]. Recent reviews have suggested that, in general, GP gatekeeping is linked with a better quality of care and lower service utilisation [4]. Yet questions persist about patient satisfaction with the model and the accuracy of gatekeepers in identifying certain conditions e.g. [4]. In the UK, some clinical commission groups have alleviated GPs of their gatekeeping responsibilities for specific clinical populations, including paediatrics and some mental health services [5]. This has been done by shifting gatekeeping duties to professionals in adjacent fields (e.g., health visitors, social care, and education) or introducing direct referral or self-referral models.

The assessment of developmental conditions such as autism spectrum disorders (autism) and attention deficit hyperactivity disorders (ADHD) reflects these broader tensions around the

For peer review only - http://bmjopen.bmj.com/site/about/guidelines.xhtml

gatekeeping role in primary care. Referral pathways in the UK often require that GPs initiate referrals for children where there is a query of autism or ADHD to Child and Adolescent Mental Health Services (CAMHS). Much of the research on autism and ADHD in general practice focuses on GP knowledge and attitudes towards the respective conditions [6-9]. Survey work indicates that in general GPs have a sound understanding of autism but little confidence responding to the condition [7]. Still, review work on GPs' knowledge of autism and ADHD have identified some outmoded aetiological theories still receiving endorsement [6 10]. Consequently, calls for training from GPs and researchers alike are recurrent in much of this work.

Remarkably few studies, however, have explored how GPs make these decisions in practice. This is within a context where parents often describe the pathway to diagnosis as challenging [11-13], and reasons for delays in referral are often felt by parents to be unclear. Some insight, however, can be gleaned from a Canadian study by Kennedy, et al. [14] on medical students at the University of Toronto, which explores knowledge-practice discrepancies following educational programmes. In this qualitative study, the authors identified various factors, including patient motivations, systemic issues, social and clinician factors as explanations for referral decisions. Increased uncertainty and urgency, somewhat predictably, prompted referrals [14]. Indeed, clinical judgment appears to be an essential factor even within contexts where best practice guidance recommends standardised screening for developmental conditions [15]. In the UK, best practice guidance[16 17] suggests that standardised tools are not essential to identify possible autism in children, and universal screening for ADHD in is explicitly discouraged. Instead, the National Institute for Health and Care Excellence (NICE) recommends that referrers, including GPs, explore possible behavioural markers, predisposing factors (e.g., family history), and obtain an account of these features across different contexts.

Our study sought to provide an account of the assessment practices some UK-based GPs engage in when identifying autism and ADHD. This research was conducted in the east of England, where changes to the configuration of local pathways mean that referrals from GPs are rarely accepted. Therefore, a subsidiary aim was to explore how GPs experience these changes and how clinicians in specialist services think about the role of GPs. Although this study takes place in a particular setting, the themes identified here might have relevance to broader national conversations about the organisation of referral pathways and the gatekeeping role of GPs.

For peer review only - http://bmjopen.bmj.com/site/about/guidelines.xhtml
For peer review only

Method

The data presented in this study were collected as part of a project exploring assessment practices in health care professionals (n=25). Specifically, we conducted semi-structured interviews with eight GPs and seven healthcare professionals working across a neurodevelopmental team and child and adolescent mental health services (CAMHS) in an English city. Additionally, we interviewed a further ten professionals who were working at various social and neurodevelopmental services, including tier 4 national services, across the UK. Here we report on the part of the study concerned with GPs' experiences of identifying autism and ADHD, changes to local referral pathways, and the views of specialists regarding the role of GPs in the neurodevelopmental assessment. This project was approved by the University of Cambridge Psychology Ethics Committee [PRE.2018.019], The Health Research Authority and local NHS research and development teams. All participants provided written informed consent before data collection. Consent was also provided verbally at the end of each interview.

Referral Pathway

The study was conducted in a socioeconomically diverse area in the East of England, in urban and rural areas serving a population of nearly a million people. Here, community and paediatric teams often work together to provide services for children under five years with a suspected developmental condition including autism and ADHD. Recent changes to the referral pathway mean that referral pathway is configured such that referrals mostly come from preschools and or health visitors, rather than GPs. For school-aged children, referrals tend to go through schools unless the child has an established neurodevelopmental condition. In the first instance, most parents are offered support in form of psychoeducation and parenting groups by neurodevelopmental team. Should questions remain about the child's development, then an assessment is conducted by the CAMHS-neurodevelopmental team. The CAMHS neurodevelopmental team is comprised of various professionals including psychologists, psychotherapists, psychiatrists, nurses, occupational therapists, speech and language therapists and paediatricians. CAMHS- community team, on the other hand, work with children with mental health problems and accept a referral from an array of sources including GPs, allied healthcare professionals, social workers, and education professionals. There are also teams specialising in child safeguarding.

For peer review only - http://bmjopen.bmj.com/site/about/guidelines.xhtml

Data collection

The local clinical research network invited GPs to take part. Professionals from CAMHS, social and neurodevelopmental services were recruited using a combination of purposive, convenience, and snowball sampling techniques. BC conducted all interviews either in person or remotely (e.g. via telephone). Face-to-face interviews were conducted in GP practices or clinic rooms. Data were collected between January and May 2019. For further information, see Table 1. Before data collection, we developed a topic guide based on existing literature and experience of the authors. The guide was piloted with three healthcare professionals working in mental health or developmental services. Questions were also discussed with two academic GPs. The final version of the guide was divided into the following sections: professional background, routine clinical work, a hypothetical case study, and referral pathways. See supplement (S1) for the hypothetical case study. The hypothetical case study and the discussions of routine clinical work were used in an effort to elicit in-depth information about clinical reasoning and assessment practices. At the beginning of each interview, participants were asked not to disclose any personally identifiable information about any patients. Questions in the section on routine clinical work were also prefaced with this reminder (see supplement S2 for interview guide).

BC has experience working in a neurodevelopmental service as an assistant psychologist, where he became interested in the interaction between cognate health services. MW is a consultant clinic psychologist and RD is a social scientist. Both RD and MW are interested in assessment practices for social and neurodevelopmental conditions. AM is a placement student with an interest in child development.

We adopted an 'information power' approach to guide recruitment and sample size [18]. This approach spotlights the following considerations for establishing a sample size in qualitative research: study aim; sample specificity; established theory; quality of dialogue; and analysis strategy [18].

Patient and Public Involvement

A general patient and public review panel at a local hospital provided feedback and suggestions on the research materials, including the topic guide. This panel did not necessarily have specific experience or personal contact with ASD or ADHD.

For peer review only - http://bmjopen.bmj.com/site/about/guidelines.xhtml

Data analysis

Data were analysed and interpreted using the framework method outlined by Gale, et al. [19]. This method has the advantage of inductive and deductive elements. This allows for ideas from the existing literature to be brought together with data derived from the interviews to develop an analytical framework. This included a recent systematic review on autism in general practice [10] and a review by Tatlow-Golden and colleagues[6] on GPs and ADHD. All interviews were transcribed by BC or a professional transcription service. Transcripts were read three times, and all audio recordings were listened to at least once before the first round of coding. In the initial stages, transcripts were coded using line-by-line coding. All transcripts were coded by BC, and several of the transcripts were also read in full by AM and RD. Regular meetings were held between the authors to discuss the data. All authors approved the final series of themes. Transcripts were coded by hand, and data were organised and grouped using flashcards. Here, results pertaining to identification and referral pathways are discussed. Specialist neurodevelopmental assessment practices and differential conceptualisation are explored elsewhere. Prior to submission, participants were each sent the results and offered the opportunity to comment on the findings.

Table 1: Participant and interview characteristics

Participant ID	Gender	Experience (years)	Setting	Interview Length
**PTGP01**	Female	>20 years	Local GP practice	43 mins
**PTGP02**	Male	4 years	Local GP practice	41 mins
**PTGP03**	Female	>20 years	Local GP practice	44 mins
**PTGP04**	Male	>20 years	Local GP practice	64 mins
**PTGP05**	Male	19 years	Local GP practice	29 mins
**PTGP06**	Male	>20 years	Local GP practice	37 mins
**PTGP07**	Male	>20 years	Local GP practice	71 mins
**PTGP08**	Male	14 years	Local GP practice	61 mins
**PTND01**	Male	17 years	Local ND service	66 mins
**PTND02**	Female	>20 years	Local ND service	64 mins
**PTND03**	Female	13 years	Local ND service	58 mins
**PTND04**	Female	> 20 years	Local ND service	64 mins
**PTND05**	Female	14 years	Local CAMHS	69 mins
**PTND06**	Female	13 years	Lifespan Autism Service	65 mins
**PTND07**	Male	3 years	Child autism service	55 mins
**PTND08**	Female	10 years	Tier 4 CAMHS	62 mins
**PTND09**	Female	19 years	Tier 4 CAMHS	53 mins
**PTND10**	Female	10 years	Tier 4 CAMHS	58 mins

For peer review only - http://bmjopen.bmj.com/site/about/guidelines.xhtml

**PTND11**	Female	16 years	Tier 4 CAMHS	48 mins
**PTND12**	Male	6 years	Tier 4 CAMHS	54 mins
**PTND13**	Female	>20 years	Tier 4 CAMHS	43 mins
**PTND14**	Male	>20 years	Tier 4 CAMHS	55 mins
**PTND15**	Female	4 years	Tier 4 CAMHS	61 mins
**PTND16**	Male	4 years	Local CAMHS	63 mins
**PTND17**	Female	>20 years	Local ND	65 mins

ND = Neurodevelopmental, CAMHS = Child and Adolescent Mental Health Services

Results

The findings are presented in two sections. The first section focuses on the methods and sources of information the GPs (n=8) used when screening possible autism and ADHD in children. The second section discusses material from the entire set of transcripts (n=25) to explore a range of perspectives on changes to the pathway and the role of the GP. A summary of the main themes is presented in Table 2.

Table 2: Summary and description of the main themes

	Themes	Description
Identification	Explicit Information	This theme describes forms of information which are considered explicit. This includes reference materials, behavioural markers, and parental report.
	Implicit Information	This theme captures forms of information which are less ostensive than material described above but nevertheless contribute to clinical decisions. This includes clinical intuition and prior knowledge of families.
Referral Pathways	Perceptions of the new referral pathway	This theme provides an account of GPs and specialists impressions of the new pathway.
	Specialist views on the role of GPs	This theme describes specialists' views on the role of GPs.
	Information sharing as a barrier and opportunity	This theme describes participant's views on information sharing between services.

For peer review only - http://bmjopen.bmj.com/site/about/guidelines.xhtml

For peer review only

Identification

There was some variation regarding the methods and techniques used by GPs to identify autism and ADHD in children. References to a diverse array of forms of information could be seen across the transcripts, including both tacit and explicit sources. These include various clinical or behavioural markers, unstructured behavioural tasks (e.g. "pointing to assess joint attention" task, prior knowledge of the family, and discussions with colleagues, and personal experience. Nevertheless, the extent to which GPs considered, used, and triangulated this information varied considerably, with some GPs offering to contact schools and others basing the referral on parental report.

Explicit information: An assortment of diagnostic or clinical markers for each condition were described by participants. Oft cited features of autism included atypical eye-contact, delayed language, fixed or specialised interests (e.g. US Emergency Departments), ritualistic behaviours (e.g. rocking), and sensory sensitives. When thinking about ADHD, most practitioners characterised the condition by inattention, problems with concentration, impulsivity, social problems, and impaired academic functioning. Yet some GPs expressed uncertainty and hesitancy when asked about particular indicators:

"Early markers? I'd probably have to look it all up, actually…And often I do. When I've got a patient coming in, I just have a sort of screen what the most common symptoms" PTGP02

"There's gonna[sic] be diagnostic criteria for that but don't ask me what they are. There's a big long list of diagnostic criteria, but I kind of think that's more a specialist job to apply the diagnostic criteria in detail before making the diagnosis, but I'd probably spot the warning signs as it were and refer on as appropriate." PTGP06

And indeed, several practitioners described looking up markers using professional sources such as GP Notebook, Clinical Knowledge Summaries or Patient.co.uk as well as some lay sources including Google or Wikipedia to find specific behavioural markers. Of note, GPs did not refer to NICE guidance.

In general, however, GPs appeared to agree on the importance of parental report. This is, of course, understandable as parental concerns are an essential component of the formal

For peer review only - http://bmjopen.bmj.com/site/about/guidelines.xhtml

assessment for many behaviourally diagnosed developmental conditions. While describing past cases, one GP commented:

“Nine-tenths is the story you’re given by the parents. Because they are the… as I say to parents, you know your son or daughter better than anybody in the world. So, we have to listen to what they have to say, [and their] ideas, concerns, and expectations” PTGP07

And indeed, the majority of participants expressed similar sentiments. Importantly, however, most GPs indicated that parental report alone was not sufficient grounds for a referral. Instead, it was suggested that such reports should be corroborated with observations of the child. Yet when facing uncertainty, approaches varied. For instance, after reflecting on complex or uncertain cases, one GP remarked:

“Just got to go with what the parents are thinking” PTGP06

However, another GP was especially concerned with diagnostic trends and the medicalisation of non-medical behaviours. For this GP, it was particularly important to triangulate parental concerns, observations of the child, and reports from the child’s school. This GP reflected on a case where parents queried a diagnosis of ADHD following conversations with a family friend:

“Speaking to the friend caused them to say maybe he [the child] has got ADHD. But in actual fact, I really don’t think he has, and the last thing you’d want is for this kid to go on unnecessary medication” PTGP04

He went on to explain that after receiving consent from the child’s parents to contact the child’s school:

“[I] spoke to his teacher and actually this was an example of where the school actually had a really good handle on him. The teacher said he’s a lovely kid, but he’s essentially feral. He just isn’t set up for rules so there was nothing he’s doing at school that would make me worried. He’s a lovely lad, and you can engage him, and he can concentrate and focus when he wants to” PTGP04

For peer review only - http://bmjopen.bmj.com/site/about/guidelines.xhtml

In contrast, however, there was a least one instance where a GP's decision to refer seemed to be based predominantly on parental insistence, rather than clinical observations or judgment:

"[Refers to another family member] seemed to know it all. [Parent] was saying that they thought the child had autism on the basis that [the child is] behind with learning, not reading and writing yet, didn't like social situations…[…]…. And they said that the school didn't think the child had autism. So, I have referred... I mean [the child] seemed normal, sat doing not a lot, but seemed normal." PTGP05

Subsequently, this participant indicated that the chances of the referral being rejected were '100%' due to the configuration of local referral pathways. When this happens, he explained he would urge the parents to go back to the school.

Tacit information: GPs also often drew implicitly from the language of folk psychology regarding typical and atypical child development. Phrases such as 'a little odd', 'just isn't what most children do" or 'clashes with normal expectations' can be found throughout the data. These were often used in reference to a specific marker or behaviour, such as "rituals and behaviours that weren't quite in keeping with a normal child of her age". Here the term tacit knowledge is used broadly to refer to practical or soft knowledge that is not easily quantifiable.

Clinical intuition was important for deciding between typical and atypical development, but at times, challenging to articulate:

"As a GP you get a subconscious idea of the spectrum of the range with children - from the kid who'll sit there like butter wouldn't melt in their mouth, like a bit oddly so, to the kid who's climbing up your curtains. [And] You get a feel of parental interaction, with 'you stop doing that now I've told you before' to the parent who just watches the child smash your ophthalmoscope'" PTGP04

"I think it's difficult, sometimes, to describe what turns into a kind of sixth sense. Really you get a clue, don't you? And sort of that kind of gut feeling, but it is about the behaviour." PTGP07

For peer review only - http://bmjopen.bmj.com/site/about/guidelines.xhtml

Prior knowledge or experience with specific children and families was also crucial for several GPs. When reflecting on cases, it was not uncommon for practitioners to preface conceptualisations with remarks such as 'I've known him since... well antenatally', 'I know the family' or '[Mum/Dad] is also my patient'. This seemed to offer a degree of context and explanation for the child's presentation. For instance, when describing children with a query of a neurodevelopmental condition, some GPs remarked on traits they had seen in other family members or diagnoses of other family members they were aware of.

GPs were also attuned to socio-environmental or parenting factors that might be contributing to the child's presenting symptoms such as discrete participating events, parental separation, or conflict. Having this overview of the patient was, for many, one of the core strengths of general practice:

"I suppose this is where Family Medicine really comes into its fore, isn't it? Because they're [both child and parents] usually, not always, but usually all our patients. So, sometimes we have this interesting dilemma about whom is the patient." PTGP07

Yet this expertise, some felt, was not always appreciated by colleagues in specialist services. When reflecting on the experience of having referrals rejected, one GP remarked:

"I sometimes wonder whether they [specialist assessment service] actually consider the family factors that we know of that we write in out letters" PTGP03

Perceptions of the new referral pathway

Most of the GPs were aware of the changes to the assessment pathway that meant referrals for neurodevelopmental assessment typically come through schools or health visitors. However, it was unclear whether two GPs were aware of these changes. Three stances to these changes were identified in the data: accepting, ambivalent, and critical. Practitioners who were more accepting of the changes tended to reason that schools are better positioned to identify such developmental conditions:

"Well, you see I think community paediatrics probably has a point. Because small child gets brought in to see the doctor and they're looking around looking reasonably normal but what do

For peer review only - http://bmjopen.bmj.com/site/about/guidelines.xhtml

I know. Whereas the school and other people that interact with the child over a long period of time are in a better position to make an assessment than me." PTGP05

"They'll [schools] be better at recognising it than me, so I'm happy, doesn't matter where the referral comes from, as long as it happens in a timely way it doesn't have to come from a GP." PTGP06

One participant had a more ambivalent attitude. For this participant, there was an acknowledgement that schools are often well-placed to identify atypical development. Still, they maintained that limiting the ability of GPs to refer put them in a challenging position:

"Difficult. Because you can see the logic in that, actually there's so much more to this than having a name put to your child's odd behaviour very few of these children will benefit from something medical…[…]… the problem comes really when a parent comes in and says I've been to the teacher three times, and the teacher says they think he's fine and if you're really worried you can go and see your GP. Because you've no idea did the teacher really say that." PTGP04

While one GP was critical of the pathway:

"Just a disaster, just a road crash really - trying to get children seen with developmental or behavioural problems is increasingly difficult, and in fact, for many patients, we end up having to go if they're school age we end up having to go through school…[…]... And that's a real nightmare for me because it means I'm having to delegate that to a third party who is not actually a health service" PTGP07

This GP felt that the pathway was also a threat to professional status and identity, reflecting a devaluation of primary care.

Professionals in the neurodevelopmental services tended to view the changes as positive. Professionals in the neurodevelopmental team reported on the impact these changes have had on service-level pressures, including waiting times for assessment:

For peer review only - http://bmjopen.bmj.com/site/about/guidelines.xhtml

"We've got the shortest waiting times for assessment for autism and ADHD. Less than eighteen weeks, whereas they were eighteen months to two to three years [before]" PTND01

Specialist views on the role of GPs

When asked directly whether GPs had a role in identifying developmental conditions, most specialists indicated that there was indeed a role for GPs. Yet this was often couched with an array of caveats about professional and organisational barriers to identification. The most common barrier, according to the specialists, was the duration of primary care consultations and a lack of training or knowledge about neurodevelopmental conditions:

"They need to be given more time to do it properly and more training. They get very little training at all really but if they got proper training and given a bit more time. Even fifteen-twenty minutes, but at the moment all they could do is to at least know the NICE guidance and know what are the signs and symptoms and take a detailed history and follow the local pathway really. Clearly, if we have GPs with a special interest in children, they got better training, and clearly, they have a lot of role to play with the ADHD medication shared care and those kinds of things." PTND01

"At best, what they should do is make good referrals to specialist teams. But beyond that, I don't know if it would be useful for people who are under massive strain and pressure and who have like whatever is it eight to ten-minute appointments, I hear that's the average, but I've never had any more than six minutes really, so I mean I don't know how you could do anything bar account for the family's request and signpost them to the appropriate teams." PTND07

A lack of training was also framed as problematic by one GP:

"I think also in terms of what we get taught. It may change now obviously. I trained thirty years ago literally we had no training at all...[…]… We'd all heard of autism but everything I know about neurodevelopmental disorders, not that there's much of it, has been acquired post-grad." PTGP04

References to the time afforded for consultations can also be found throughout the GP interviews. For some, this was felt to be a significant barrier to identification. To

For peer review only - http://bmjopen.bmj.com/site/about/guidelines.xhtml

circumnavigate some of these challenges, one GP described bringing families back for multiple consultations.

Information sharing as a barrier and opportunity:

Another topic that runs through the data is the importance of informal networks and the issues with sharing information between services. In general, informal networks could be described as internal and external. Internal networks mostly consisted of practice staff, including administrative staff, GP colleagues and nurses. By contrast, external networks consisted of educational professionals and colleagues in secondary care. Due to the reconfiguration of primary care services, health visitors seemed to occupy a position between these two networks:

"We used to have Health Visitors attached to the practice, but they don't exist anymore. I don't know who our Health Visitor is. I've never met them." PTGP05

Meanwhile, most GPs acknowledged that nursery staff, primary school teachers and other educational professionals were essential sources of information when thinking about child development. Yet the lack of a linked system for educational and primary care records presented challenges in terms of sharing this information:

"We have occasional contact with schools but not very much. Not often. I'd be unsure about the boundaries and confidentiality and things like that, to be honest." PTGP05

There also seemed to be a lack of communication between GPs and specialist services:

"It's so difficult because you know you'll write the letter, but you don't know if they'll actually get any help or whether they'll get put on the waiting list or whether someone else will monitor the child. So that's the tricky bit, really." PTGP03

"let's say we're querying autism they [neurodevelopmental team] would send the referral back. And say it needs to be referred through the school which is quite doable because quite often they have started with the school. And the school have said have you seen your GP and of course then it looks like passing the parcel." PTGP04

For peer review only - http://bmjopen.bmj.com/site/about/guidelines.xhtml

Discussion

Summary

GPs used tacit and explicit forms of information when identifying autism and ADHD in children. These included clinical or behavioural markers, parental report, prior knowledge of the child and family, and professional networks. For most, parental concerns were the chief factor driving referral decisions. However, a few participants described instances where they had sought information from other sources (e.g. schools). Nevertheless, changes to the configuration of local pathways have meant that referrals from GPs for neurodevelopmental assessment are now rarely accepted. GPs had mixed views on these changes. Most specialists agreed that GPs did have a role in identifying neurodevelopmental conditions yet expressed concerns about a perceived lack of training or knowledge and framed time pressures as problematic.

Strengths and Limitations

The current study adds to our understanding of early identification by gleaning the perspectives of GPs and those in specialists' assessment services. From a methodological perspective, the flexible interview guide and the combination of case-based discussions and hypothetical case study allowed us to elicit rich narratives about these topics. Further, by analysing discourses of past and hypothetical cases, we were able to explore some of the other forms of knowledge that come into play. Additionally, our study was conducted in a setting where GPs have been, to a large extent, absolved of their gatekeeping responsibilities for identifying autism and ADHD in children. Therefore, the current study presents a unique opportunity to explore how GPs experience having a reduced role for a specific patient group and thus adds to national conversations about the nature and future of general practice. That said, it is essential to consider whether the findings about identification are transferable to other contexts. Regarding identification, given that specialists espoused similar issues with referrals in different settings, it seems unlikely that the methods and techniques used by GPs in this area were atypical. As recruitment of GPs was completed through the local CRN, it is not possible to determine how many GPs decided not to take part in the study. This might raise other concerns about the representativeness of the GP sample. However, as the analysis illustrates, there was considerable diversity in the views and opinions expressed by the GPs. Another limitation of this study is that, although data were discussed at regular meetings between the research group, BC conducted and coded the analysis. As per the method, BC has previously worked in a neurodevelopmental service. To address possible issues with research bias, BC wrote

For peer review only

reflections throughout the process and engaged in peer and academic supervision. Finally, this research took place prior to Covid-19 pandemic. Therefore, with GPs under considerable strain, it is important to consider whether how the pandemic might have shaped referral pathways and indeed GP's attitudes towards identifying neurodevelopmental conditions.

Comparison with the literature

Most studies about GP knowledge of autism and ADHD have focused on explicit knowledge of clinical markers [6 7 9 20]. Yet, as others have shown, clinical judgment is core to referral decisions [14 21]. Naturally, knowledge of clinical markers is important for identifying these conditions. For ADHD, some co-developed training tools are showing promise [22]. Still, an overemphasis on this form of knowledge risks driving attention away from the other sources GPs draw on, including prior experience with the child or family. Our study, therefore, adds to the understanding of identification by tracing out the various forms of explicit and tacit material which GPs draw upon when determining whether a child requires formal assessment.

Several studies have identified that GPs frequently report having little training in autism e.g. [7] and ADHD [6]. It follows that more training could be helpful. Our data lend some support to these findings, and broadly speaking, we agree with these calls for more training. The 'lack of training' thread runs throughout the primary care literature. However, a degree of caution is warranted, as framing the problem as one of 'a lack of training' risks a) flattening the conceptual complexity associated with identifying these conditions b) silencing the host of organisational shortcomings that make referral decisions challenging, and c) camouflaging alternate solutions such as the integration of health, educational, justice or social care records or changing pathways.

Implications for Research and Practice

Elsewhere, questions have been raised about GP gatekeeping [1-3]. As such, zooming in on a particular pathway means that we were able to explore in detail how those on the ground experienced changes to GP gatekeeping. It might be envisaged that GPs would welcome changes that reduce some of the pressure on them. Yet GPs in this study expressed mixed views. In contrast, specialists tended to view the changes positively and credit these changes with preventing saturation of the service. Our research is not positioned to explore the impact that these changes have on service delivery. We recommend that future work explores how such changes impact patient satisfaction, waiting lists, and numbers of accepted referrals. It

For peer review only - http://bmjopen.bmj.com/site/about/guidelines.xhtml

will be also important to consider the unmet needs of children who do not receive access to services.

Issues around the quality of GP referrals ran through the specialist interviews. As such, we anticipate that the analysis of autism and ADHD referrals using health records might yield further insights into the level and quality of information required by specialist services.

Finally, it seems likely that GPs in most settings will retain gatekeeping responsibilities for autism and ADHD for the foreseeable future. The findings indicate that some GPs used lay sources such as Google or Wikipedia. As such, we recommended that future work further explores the modes of professional and lay information used by GPs to inform their clinical decision making. In particular, we would welcome research that explores whether the forms of information used by GPs has an impact on referral decisions and on referral acceptance.

For peer review only

a. Contributorship statement

BC, MW and RD contributed to the conceptualisation and design of the study. BC applied for governance and ethical approvals, and collected the data. BC conducted the initial coding while RD, AM and MW contributed to analysis of the data. Each author offered interpretations of the findings. The final set of themes were agreed by each of the authors. BC wrote the first draft of the manuscript. MW, AM and MW provided critical feedback and suggestions on subsequent drafts. All authors contributed to and approved the final manuscript.

b. Competing interests

None to Declare

c. Funding

The authors wish to thank NIHR School for Primary Care Research [RG94577] for their support for work on this paper. This research was also funded in whole, or in part, by the Wellcome Trust [WT103343MA]. For the purpose of open access, the author has applied a CC BY public copyright licence to any Author Accepted Manuscript version arising from this submission.' We would also like to the CRN for help with recruitment. Warm thanks are extended to authors Prof Marinus van IJzendoorn for feedback on a draft of the manuscript. The views expressed are those of the authors and not necessarily those of the CRN, Wellcome, NHS, the NIHR or the Department of Health.

d. Data sharing statement

Although all participants were reminded not to disclose any personally identifiable information about patients or families, the transcripts do include reflections on routine clinical work and service arrangements. Thus, to further safeguard the privacy or the participants and those involved in their services, we cannot make the transcripts available. Please contact the authors for further details on the data.

Ethics Statement

For peer review only - http://bmjopen.bmj.com/site/about/guidelines.xhtml

This project was approved by the University of Cambridge Psychology Ethics Committee [PRE.2018.019].

For peer review only

For peer review only - http://bmjopen.bmj.com/site/about/guidelines.xhtml

References

1. Forrest CB. Primary care in the United States: primary care gatekeeping and referrals: effective filter or failed experiment? *BMJ* 2003;326(7391):692-5. doi: 10.1136/bmj.326.7391.692 [published Online First: 2003/03/29]
2. Franks P, Clancy CM, Nutting PA. Gatekeeping revisited—protecting patients from overtreatment. *New England Journal of Medicine* 1992;327(6) doi: 10.1056/nejm199208063270613
3. Vedsted P, Olesen F. Are the serious problems in cancer survival partly rooted in gatekeeper principles? An ecologic study. *The British journal of general practice : the journal of the Royal College of General Practitioners* 2011;61(589):e508-12. doi: 10.3399/bjgp11X588484 [published Online First: 2011/08/02]
4. Sripa P, Hayhoe B, Garg P, et al. Impact of GP gatekeeping on quality of care, and health outcomes, use, and expenditure: a systematic review. *The British journal of general practice : the journal of the Royal College of General Practitioners* 2019;69(682):e294-e303. doi: 10.3399/bjgp19X702209 [published Online First: 2019/03/27]
5. Greenfield G, Foley K, Majeed A. Rethinking primary care's gatekeeper role. *BMJ* 2016;354:i4803. doi: 10.1136/bmj.i4803 [published Online First: 2016/09/25]
6. Tatlow-Golden M, Prihodova L, Gavin B, et al. What do general practitioners know about ADHD? Attitudes and knowledge among first-contact gatekeepers: systematic narrative review. *BMC family practice* 2016;17(1):129. doi: 10.1186/s12875-016-0516-x
7. Unigwe S, Buckley C, Crane L, et al. GPs' confidence in caring for their patients on the autism spectrum: an online self-report study. *The British journal of general practice : the journal of the Royal College of General Practitioners* 2017;67(659):e445-e52.
8. Kirby A, Davies R, Bryant A. Do teachers know more about specific learning difficulties than general practitioners? *British Journal of Special Education* 2005;32(3):122-26.
9. Garg P, Lillystone D, Dossetor D, et al. An exploratory survey for understanding perceptions, knowledge and educational needs of general practitioners regarding autistic disorders in New South Wales (NSW), Australia. *Journal of clinical and diagnostic research: JCDR* 2014;8(7):PC01.
10. Coughlan B, Duschinsky R, O'Connor M-E, et al. Identifying and managing care for children with autism spectrum disorders in general practice: A systematic review and narrative synthesis. *Health & Social Care in the Community* 2020;28(6):1928-41. doi: 10.1111/hsc.13098
11. DosReis S, Barksdale CL, Sherman A, et al. Stigmatizing experiences of parents of children with a new diagnosis of ADHD. *Psychiatr Serv* 2010;61(8):811-6. doi: 10.1176/ps.2010.61.8.811 [published Online First: 2010/08/03]
12. Ryan S, Salisbury H. 'You know what boys are like': pre-diagnosis experiences of parents of children with autism spectrum conditions. *The British journal of general practice : the journal of the Royal College of General Practitioners* 2012;62(598):e378-e83.
13. Boshoff K, Gibbs D, Phillips RL, et al. A meta-synthesis of how parents of children with autism describe their experience of advocating for their children during the process of diagnosis. *Health & Social Care in the Community* 2019;27(4):e143-e57. doi: 10.1111/hsc.12691

For peer review only

14. Kennedy T, Regehr G, Rosenfield J, et al. Exploring the Gap Between Knowledge and Behavior: A Qualitative Study of Clinician Action Following an Educational Intervention. *Academic Medicine* 2004;79(5):386-93.
15. Hyman SL, Levy SE, Myers SM. Identification, Evaluation, and Management of Children With Autism Spectrum Disorder. *Pediatrics* 2020;145(1) doi: 10.1542/peds.2019-3447 [published Online First: 2019/12/18]
16. National Institute for Health and Care Excellence. Autism spectrum disorder in under 19s: recognition, referral and diagnosis Clinical guideline [CG128] 2017 [Available from: https://www.nice.org.uk/guidance/cg128/chapter/Recommendations#after-referral-to-the-autism-team.
17. National Institute for Health and Care Excellence. Attention deficit hyperactivity disorder: diagnosis and management NICE guideline [NG87] 2019 [Available from: https://www.nice.org.uk/guidance/ng87/chapter/Recommendations#recognition-identification-and-referral accessed Jan 5th 2022.
18. Malterud K, Siersma VD, Guassora AD. Sample Size in Qualitative Interview Studies: Guided by Information Power. *Qual Health Res* 2016;26(13):1753-60. doi: 10.1177/1049732315617444 [published Online First: 2015/11/29]
19. Gale NK, Heath G, Cameron E, et al. Using the framework method for the analysis of qualitative data in multi-disciplinary health research. *BMC Medical Research Methodology* 2013;13(1):117. doi: 10.1186/1471-2288-13-117
20. Shaw K, Wagner I, Eastwood H, et al. A qualitative study of Australian GPs' attitudes and practices in the diagnosis and management of attention-deficit/hyperactivity disorder (ADHD). *Family Practice* 2003;20(2):129-34. doi: 10.1093/fampra/20.2.129
21. Thomas R, Spragins W, Mazloum G, et al. Rates of detection of developmental problems at the 18-month well-baby visit by family physicians' using four evidence-based screening tools compared to usual care: a randomized controlled trial. *Child: care, health and development* 2016;42(3):382-93. doi: 10.1111/cch.12333
22. French B, Daley D, Perez Vallejos E, et al. Development and evaluation of an online education tool on attention deficit hyperactivity disorder for general practitioners: the important contribution of co-production. *BMC Family Practice* 2020;21(1):224. doi: 10.1186/s12875-020-01289-5

For peer review only

For peer review only - http://bmjopen.bmj.com/site/about/guidelines.xhtml

Case Study 1:

Reception received a phone call from patients Linda (33) and Tim (32) regarding an appointment for their son, Robert (6). The family are known to the practice and previously there have been safeguarding concerns and social services have been involved with the family.

In the initial phone call, Linda requested the next available appointment with the GP. They were subsequently booked in for an appointment in two weeks' time. The next day, Tim phoned reception to express his dissatisfaction with the waiting list and requested that they be given priority in the event of a cancellation.

Two weeks later Linda, Tim, and Robert arrived for the appointment. From the outset, Robert appeared distressed (i.e. crying). Linda made numerous attempts to comfort Robert, but he moved away in response to each of her approaches. At one-point Robert kicked out at Linda. There are what look like two distinctive episodes of hand-flapping.

As the consultation progressed, Robert gradually became more comfortable and was very active (e.g. jumping around the room). He moved from one activity to another in quick succession. Robert's eye contact was fleeting, and seemed to have a restricted range of facial expressions. In terms of conversation, Robert spoke in complex sentences, although the subject matter was a little repetitive and mainly around his favourite toy (Shopkins). Tim then took Robert to the waiting room, so Linda could discuss their concerns with the GP.

According to Linda, Robert has few friends in school and teachers are concerned about his academic progress. Additionally, Robert has become increasingly aggressive towards her and recently threw her laptop at a wall. In terms of history, she reports no significant issues with birth or pregnancy. Robert achieved his motor milestones; however, his language development was delayed. Previously, he received speech and language therapy in the community. Robert has an older half sibling, Chris (14) who has a diagnosis of ADHD. When asked about development prior to three years, Linda disclosed that Robert lived with his grandmother beginning when he was 18 months old to just after his third birthday, as Linda and Tim were separated during this period. During the separation, Linda was an inpatient at a local mental health facility.

For peer review only

Supplement 2. Full study interview guide

Introduction:

I would like to ask you some questions about your training and background, your routine clinical activities, and your experiences and views on the diagnostic process for developmental conditions in children. I would like to remind you not to disclose any personally identifiable information about any individuals, child or families you work with during the interview.

Background
Can you give me a brief overview of your current position?
Probe for experience related to child development

How long have you been working as a (GP, psychologist, psychiatrist etc)?
Probe for length of time working with children and families

Where and when did you do your clinical training?
Probe for General Practitioner (GP) training vs medical training

Clinical work

Without revealing any personally identifiable information, can you tell me about a case where you conceptualised the child's features as relating to autism?
Probe action steps
Probe specific difficulties with working with this child
Probe for informal assessment (e.g. familiarity with things in the past)
Probe assessments (I know X is saturated with screening tools/assessments; observations)
Probe for level of clarity (how certain or uncertain were you about X)
Repeat for ADHD and attachment related

If not clear, can you give me an overview of your involvement in relation to identification and assessment of developmental (or behavioural) difficulties?
Probe for assessment tools, observations and focus on developmental histories, team-based approach etc.
If GP, probe for screening tools.

Can you think of a specific instance where there was uncertainty regarding the nature of a child's difficulties/diagnosis?
Probe for description
Probe for action steps

Are there other factors that contributed/routinely contribute to the decision you made (e.g. system factors, availability of supports and services)?
Have you ever been involved with overturning or changing a child's diagnosis?

Case Study Questions

For peer review only

Questions for participants with Case Study 1,

What are your initial thoughts on the case?
What questions would you ask A) Robert B) Parents (Tim & Linda)
What would you advise as the next steps?
If the participant deems onward referral is necessary
what information would you put in the referral letter?
Why did X resonate with you?
How easy have you found it to get external help

What did you find yourself drawing on to make sense of Roberts presenting difficulties? (i.e. other similar cases, diagnostic criteria, theory etc)

If the child had a private diagnosis of autism, would that influence how you think about the case?

If you were unsure about the nature of the child's difficulties, are there other professionals you would consult with?

Questions for participants with Case study 2.

What are your initial thoughts on the case?
Probe for possible diagnosis, working clinical hypotheses, formulation

If the participant states a specific diagnosis/conceptualisation
What features of the case helped you to arrive at that conclusion?
Are there other conditions you considered?
Probe, if so what?
Probe how the participant differentiated

If the participant indicates that it could be multiple conditions
You mentioned that it could be X or Y, how would you differentiate?
What assessments/sources of information would be useful in helping you to reach your decision?

If the participant indicates there isn't enough information to make the decision
What information would help you make a decision regarding the nature of the child's difficulties?
Are there specific assessments/ theories you would use?

In your view, is there need for further assessment?
If Yes, what are the next steps in terms of assessment?

Probe for risk assessment
Probe cognitive assessment
Probe sources of information

Are there any frameworks/ theories you used to understand the nature of the child's presenting difficulties?

For peer review only

Probe for specific models/ theories
Probe for influential book/text/ or talks

If the child had a private diagnosis of autism, would that influence how you think about the case?

If you were unsure about the nature of the child's difficulties, are there other professionals you would consult with?
Referral Pathways Information

In your experience, what do you consider the early markers of autism?
Probe for symptoms, features, predisposing events, comorbid conditions, family history
**repeat for ADHD, Attachment related difficulties.

Rotate order each interview

Questions for Primary Care Clinicians
Have you ever referred a child to a CAMHS or relevant assessment service as you suspected the child may a developmental condition or behavioural difficulty?
If yes, what information did you include in your referral letter?
Have you ever had a referral of this nature not accepted in the first instance?
If yes, why was the referral not accepted?
On a scale of 1-10, how easy have you found it to get formal assessment for a child you think refer a child who you think may have autism?
Repeat for attachment problems
Repeat for ADHD
Have you experienced any challenges to referring a child who you think may have autism?
Repeat for attachment problems
Repeat for ADHD
What, if anything could be done to make the referral pathway, clearer?
On a scale of 1-10, how confident do you feel that you will be able to get the appropriate a) assessment b) support for a child with autism, adhd, attachment problems.

Questions for non-primary care clinicians
Do you receive many referrals to your service from GPs?
If yes, how would you describe the quality of these referrals?
Probe what's in a good/less good referral
If no, where do you get the majority of referrals from
How would you describe the quality of these referrals?
What information would constitute a sufficient referral (i.e. allows you to make your decision regarding acceptance for subsequent assessment/support)
What information could be collected by a GP to assist with the decision to accept or reject a referral to your service?
How do you think children and families experience the journey from primary care to your service?
Thinking about conditions with overlapping features, do you see value in differentiating autism from attachment-related conditions?
Do you see a meaningful distinction between the attachment disorders (RAD and Disinhibited) and attachment-related difficulties?

For peer review only

Similarly, when considering a diagnosis of autism and adhd, what value do you see in diagnosing both or trying to differentiate?
Finally, what in you view is the value in differentiating ADHD from attachment-related conditions?

For peer review only

For peer review only

Standards for Reporting Qualitative Research (SRQR)*

http://www.equator-network.org/reporting-guidelines/srqr/

Title and abstract

	Page/line no(s).
**Title** - Concise description of the nature and topic of the study Identifying the study as qualitative or indicating the approach (e.g., ethnography, grounded theory) or data collection methods (e.g., interview, focus group) is recommended	Page 3/ line 1/2
**Abstract** - Summary of key elements of the study using the abstract format of the intended publication; typically includes background, purpose, methods, results, and conclusions	Page 3/ line 4

Introduction

**Problem formulation** - Description and significance of the problem/phenomenon studied; review of relevant theory and empirical work; problem statement	Page 4/ line 22
**Purpose or research question** - Purpose of the study and specific objectives or questions	Page 5/line 10

Methods

**Qualitative approach and research paradigm** - Qualitative approach (e.g., ethnography, grounded theory, case study, phenomenology, narrative research) and guiding theory if appropriate; identifying the research paradigm (e.g., postpositivist, constructivist/ interpretivist) is also recommended; rationale**	NA
**Researcher characteristics and reflexivity** - Researchers' characteristics that may influence the research, including personal attributes, qualifications/experience, relationship with participants, assumptions, and/or presuppositions; potential or actual interaction between researchers' characteristics and the research questions, approach, methods, results, and/or transferability	Page 6/ line 28
**Context** - Setting/site and salient contextual factors; rationale**	Page 5//6
**Sampling strategy** - How and why research participants, documents, or events were selected; criteria for deciding when no further sampling was necessary (e.g., sampling saturation); rationale**	Page 6 13/14
**Ethical issues pertaining to human subjects** - Documentation of approval by an appropriate ethics review board and participant consent, or explanation for lack thereof; other confidentiality and data security issues	Page 5/ line 23
**Data collection methods** - Types of data collected; details of data collection procedures including (as appropriate) start and stop dates of data collection and analysis, iterative process, triangulation of sources/methods, and modification of procedures in response to evolving study findings; rationale**	Page 5 line 16

For peer review only

**Data collection instruments and technologies** - Description of instruments (e.g., interview guides, questionnaires) and devices (e.g., audio recorders) used for data collection; if/how the instrument(s) changed over the course of the study	Page 6 line 21
**Units of study** - Number and relevant characteristics of participants, documents, or events included in the study; level of participation (could be reported in results)	Page 7 Table 1
**Data processing** - Methods for processing data prior to and during analysis, including transcription, data entry, data management and security, verification of data integrity, data coding, and anonymization/de-identification of excerpts	Page 7 line 11
**Data analysis** - Process by which inferences, themes, etc., were identified and developed, including the researchers involved in data analysis; usually references a specific paradigm or approach; rationale**	Page 7 line 6
**Techniques to enhance trustworthiness** - Techniques to enhance trustworthiness and credibility of data analysis (e.g., member checking, audit trail, triangulation); rationale**	Page 7

Results/findings

**Synthesis and interpretation** - Main findings (e.g., interpretations, inferences, and themes); might include development of a theory or model, or integration with prior research or theory	Page 8 - 15
**Links to empirical data** - Evidence (e.g., quotes, field notes, text excerpts, photographs) to substantiate analytic findings	Page 8-15

Discussion

**Integration with prior work, implications, transferability, and contribution(s) to the field** - Short summary of main findings; explanation of how findings and conclusions connect to, support, elaborate on, or challenge conclusions of earlier scholarship; discussion of scope of application/generalizability; identification of unique contribution(s) to scholarship in a discipline or field	Page 15 line 7, Page 16 starting line 5
**Limitations** - Trustworthiness and limitations of findings	

Other

**Conflicts of interest** - Potential sources of influence or perceived influence on study conduct and conclusions; how these were managed	Page 2 line 1
**Funding** - Sources of funding and other support; role of funders in data collection, interpretation, and reporting	Page 1 line 16

*The authors created the SRQR by searching the literature to identify guidelines, reporting standards, and critical appraisal criteria for qualitative research; reviewing the reference lists of retrieved sources; and contacting experts to gain feedback. The SRQR aims to improve the transparency of all aspects of qualitative research by providing clear standards for reporting qualitative research.

For peer review only
For peer review only - http://bmjopen.bmj.com/site/about/guidelines.xhtml

**The rationale should briefly discuss the justification for choosing that theory, approach, method, or technique rather than other options available, the assumptions and limitations implicit in those choices, and how those choices influence study conclusions and transferability. As appropriate, the rationale for several items might be discussed together.

Reference:

O'Brien BC, Harris IB, Beckman TJ, Reed DA, Cook DA. **Standards for reporting qualitative research: a synthesis of recommendations.** *Academic Medicine*, Vol. 89, No. 9 / Sept 2014 DOI: 10.1097/ACM.0000000000000388

For peer review only

For peer review only - http://bmjopen.bmj.com/site/about/guidelines.xhtml
